# Aryl hydrocarbon receptor in the kidney regulates metabolic cross-talk with the liver and gut microbiome

**DOI:** 10.1038/s41598-026-44083-6

**Published:** 2026-03-24

**Authors:** Neema Jamshidi, Sanjay K. Nigam

**Affiliations:** 1 Department of Radiological Sciences, University of California, Los Angeles, CA USA; 2 Institute of Engineering in Medicine, University of California, San Diego, La Jolla, CA USA; 3 Departments of Pediatrics and Medicine (Nephrology), University of California, San Diego, La Jolla, CA USA

**Keywords:** Biochemistry, Diseases, Nephrology

## Abstract

Aryl hydrocarbon receptor (AHR) is central to inter-organ and inter-organismal crosstalk along the gut microbiome-liver-kidney axis. Studies in the kidney indicate this is due its regulation of transporter-mediated "remote sensing and signaling" via small molecules derived from the gut microbiome. These include gut microbiome-derived uremic toxins (e.g., indoxyl sulfate) associated with chronic kidney disease (CKD), which are transported by OAT1 and other SLC transporters. However, how kidney AHR regulates gut microbiome-liver-kidney crosstalk is largely unexplored. Here, we applied formal multi-organ metabolic reconstruction to multiomics data from the kidney of AHR knockout mouse and contextualized it with systemic metabolic changes. Consistent with the Remote Sensing and Signaling Theory, the explicit and quantitative multi-organ host and microbe network reconstruction revealed that kidney AHR regulates correlated sets of biochemical reactions at the organ, cellular, and organellar levels. In the absence of kidney AHR, there is up-regulation of polyamine related metabolism and down regulation of pathways involving organic acids (oxalate), thiamine, and amino-, methyl-, and phospho-transferases. Importantly, these AHR-dependent changes in correlated reaction sets occur not only in the kidney but also in the liver and microbiome. The normal inter-organ and inter-organismal (host-microbe) communication via these biochemical pathways between the kidney and other organs is disrupted when AHR function is lost. Thus, the incorporation of tissue and plasma metabolomics with kidney and liver transcriptomics in a biologically coherent framework provides a novel view into the metabolic relationships across the gut microbiome-liver-kidney axis. Through a concordance–discordance assessment of the transcriptome and metabolome, we were able to identify potential metabolic regulatory hubs dependent upon kidney AHR, a uremic toxin sensor and drug target. This included observed up-regulation of urea cycle enzymes with down-regulation of more distant enzymes (that are not directly involved with nitrogen handling). The results show how AHR-dependent remote sensing and signaling can involve interactions via biochemical reaction sub-networks between distinct organellar compartments of the communicating kidney and liver.

**Supplementary Information:**

The online version contains supplementary material available at 10.1038/s41598-026-44083-6.

## Introduction

The aryl hydrocarbon receptor (AHR) is probably best known as a xenobiotic sensor^1–4^. This ligand-activated nuclear receptor regulates the transcription of a wide range of genes, including proteins involved in drug handling, including multi-specific transporters (e.g., OAT1) and drug metabolizing enzymes (DMEs)^1,5^. More recently, its roles in key endogenous metabolic processes and the immune response have been recognized^6–8^. The expression of AHR is quite widespread including the hepatobiliary, genitourinary, gastrointestinal, and immune systems and is particularly important in barrier tissue function^9^.

The kidney is one of the major organs involved in the handling of xenobiotics, including drugs and toxins. In recent years, evidence has been building for its essential role in sensing and elimination of gut microbe derived-endogenous small molecules that act as uremic toxins in chronic kidney disease (CKD). These uremic toxins, which are highly protein bound, are not easily eliminated by hemodialysis and are thought to both contribute to the malaise of patients on hemodialysis and the progression of renal and cardiovascular disease in CKD^10–13^. Gut microbiome-derived uremic toxins are often organic anions that enter the proximal tubule of the kidney by the organic anion transporters (e.g. OAT1 or SLC22A6, OAT3 or SLC22A8) and are believed to bind and activate AHR. This is particularly true for tryptophan derivatives such as indoxyl sulfate, indole propionic acid, and kynurenine^14–17^.

The gut microbiome-derived uremic toxins that activate kidney AHR cause multi-organ dysfunction along the gut microbiome-liver-kidney axis. In the kidney, AHR is increasingly believed to serve as an integrator of organ crosstalk along the gut microbiome-liver-kidney axis mediated by small organic molecules in a manner similar to what has been worked out for FXR in bile acid regulation the liver^18^. These concepts are more fully described in the Remote Sensing and Signaling Theory (RSST), which emphasizes how transporters and ligand-activated transcription factors regulate inter-organ and inter-organismal (e.g. microbiome-host) communication by small organic molecules^12,19,20^.

**Fig. 1:**
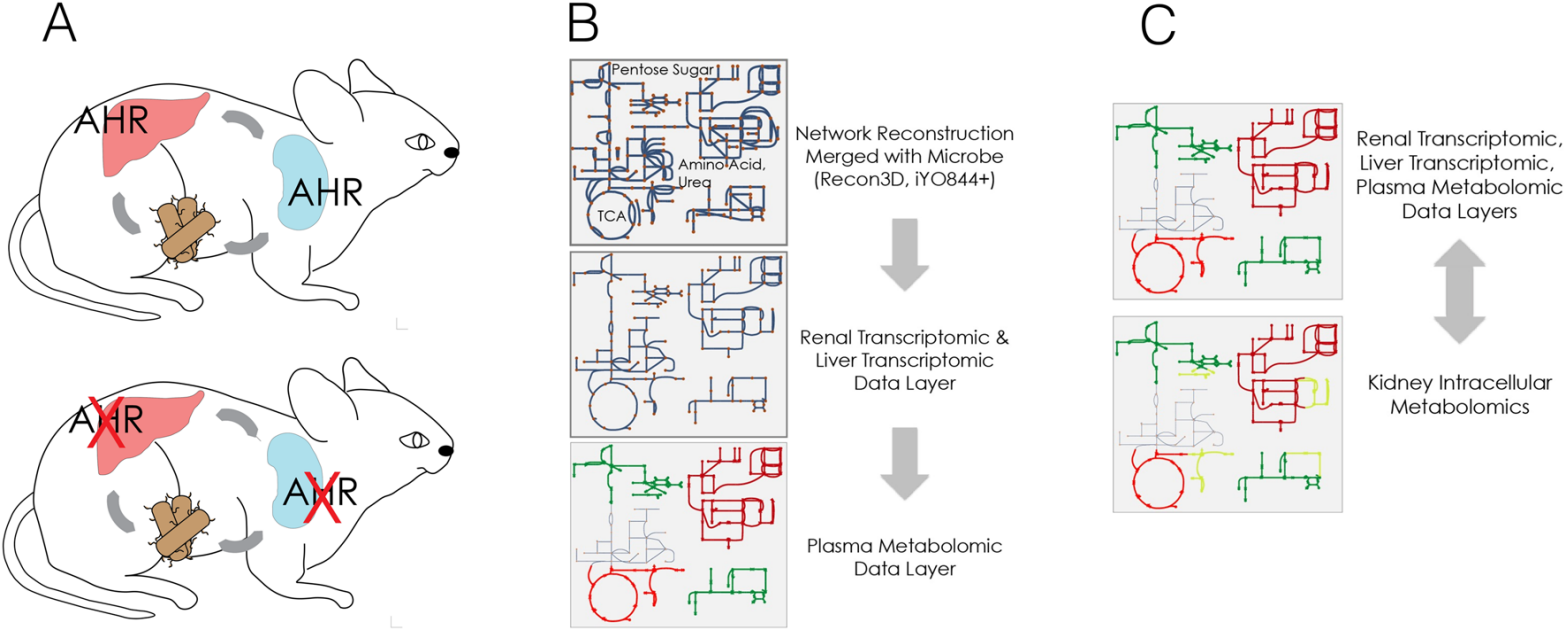
Integrative multi-organ, multi-omics metabolic reconstruction analysis. Given the broad metabolic changes observed with tissue-specific differences, there was a need to analyze changes in metabolic states in the AHR KO at multiple spatial levels: systemic, organ-specific, and organelle specific. (**A**) Experimental design. Wildtype and AHR kidney knockout mice were compared using liver, kidney, and plasma omics profiling. (**B**) High-level schematic overview of the modeling approach. A functional, multi-omics, multi-organ, constraint-based modeling approach was employed. Integrative analysis of liver transcriptomics, kidney transcriptomics, kidney metabolomics, and plasma metabolomics in AHR knockout versus wildtype mice was used to construct multi-organ metabolic reconstructions (MOMRs). The prior context is a systemic (not kidney) analysis of AHR loss^34^. Tissue specific models were then merged with a representative microbiome model. (**C**) Data-driven, context specific models enabled an integrated representation of multi-organ, multi-omics data. The resulting model enabled functional comparisons based on calculation of different flux states in the AHR knockout compared to wildtype animals accounting for the liver, kidney, and microbiome metabolic function.

Thus, there is considerable interest in characterizing the physiological function of AHR in the kidney, *per se*, compared to the whole body. We have obtained metabolomics data from the kidney of the AHR knockout mouse and analyzed it together with transcriptomic changes using genome-scale metabolic network reconstructions (GEM)^21–24^. GEM provide a ‘context for content’, in which the biological relationships between gene transcripts, the corresponding translated enzymes, and associated small metabolite substrates/products are reflected in ‘gene-protein-reaction’ (GPR) relationships^23,24^. The network reconstructions are built upon experimentally validated, substrate-specific enzymatic reactions, stoichiometric balances, transporters, and intracellular localization^25–27^. Simulations are carried out at the level of the metabolites as inter- and intra-cellular, organelle-specific biochemical reactions, and pathways. The resulting parameter-free systems approach provides a functional interpretation of the data by applying the data as “layers” of constraints. With knockout mice multi-omics data, we have previously shown this approach provides deep insights into the alterations in endogenous metabolism associated with regulation of renal function, particularly that involving drug transporters like OAT1 and OAT3^19^. Here, we further expand upon this framework to include inter-organ (liver-kidney) and inter-species (host-microbiome) interactions in constructing condition and context-specific Multi-Organ Metabolic Reconstructions (MOMR) to also account for the hierarchy of metabolic interactions in wildtype versus AHR knockout mice (Fig 1).

The results from these MOMR indicate that kidney AHR regulates biochemical reactions at multiple scales in the kidney (organ, cellular, and organellar levels). Most notably, these AHR-dependent changes in correlated reaction sets went beyond the kidney to the liver and microbiome. Thus, basal organ crosstalk and inter-organismal (host-microbe) communication involving these sets of correlated biochemical reactions is disrupted when AHR function is lost. In other words, there is ongoing remote communication along the gut microbiome-liver-kidney axis dependent on normal kidney AHR function; this is disrupted in the AHR knockout mice. Furthermore, through an evaluation of concordant and discordant analysis of the different types of multi-omics data, we were able to identify potential metabolic regulatory hubs dependent upon kidney AHR.

Because AHR appears to be critical for uremic toxin-mediated remote sensing and signaling in the proximal tubule in CKD^20,28–31^, and because this ligand-activated receptor is an important pharmaceutical target, these studies help define what types of biochemical pathways are altered across the kidney-liver-microbiome by therapies aimed at interfering with AHR function.

## Results

We focus on how AHR in the kidney regulates biochemical reactions and pathways across the gut microbiome-liver-kidney axis. Our approach combines a host model with a microbiome model to enable building a MOMR. It provides a formal quantitative portrait of remote communication via sets of biochemical reactions that interact across multiple scales in the gut microbiome-liver-kidney axis. Importantly, these quantitative analyses extend down to the level of organelle biochemistry.

Our strategy has been to analyze the multi-organ transcriptomic together with plasma and kidney metabolomic data from the wild type compared to the AHR knockout mouse using genome-scale metabolic reconstruction. This enables multi-level assessment of the metabolic alterations at the organ, cell, and intracellular levels. The resulting models enable functional comparisons based on calculation of different flux states in the AHR knockout kidney compared to wildtype animals accounting for liver, kidney, and microbiome metabolism.

The functional, integrative analysis described below enables multiple types comparisons to be made between WT and AHR knockouts, including 1) content (compartment specific metabolites, biochemical transformation enzymes, and transporter channels), 2) metabolic network structural composition, and 3) functional, flux-based analyses and simulations.

### AHR plasma metabolome disruption implicates multi-organ metabolic processes

**Fig. 2:**
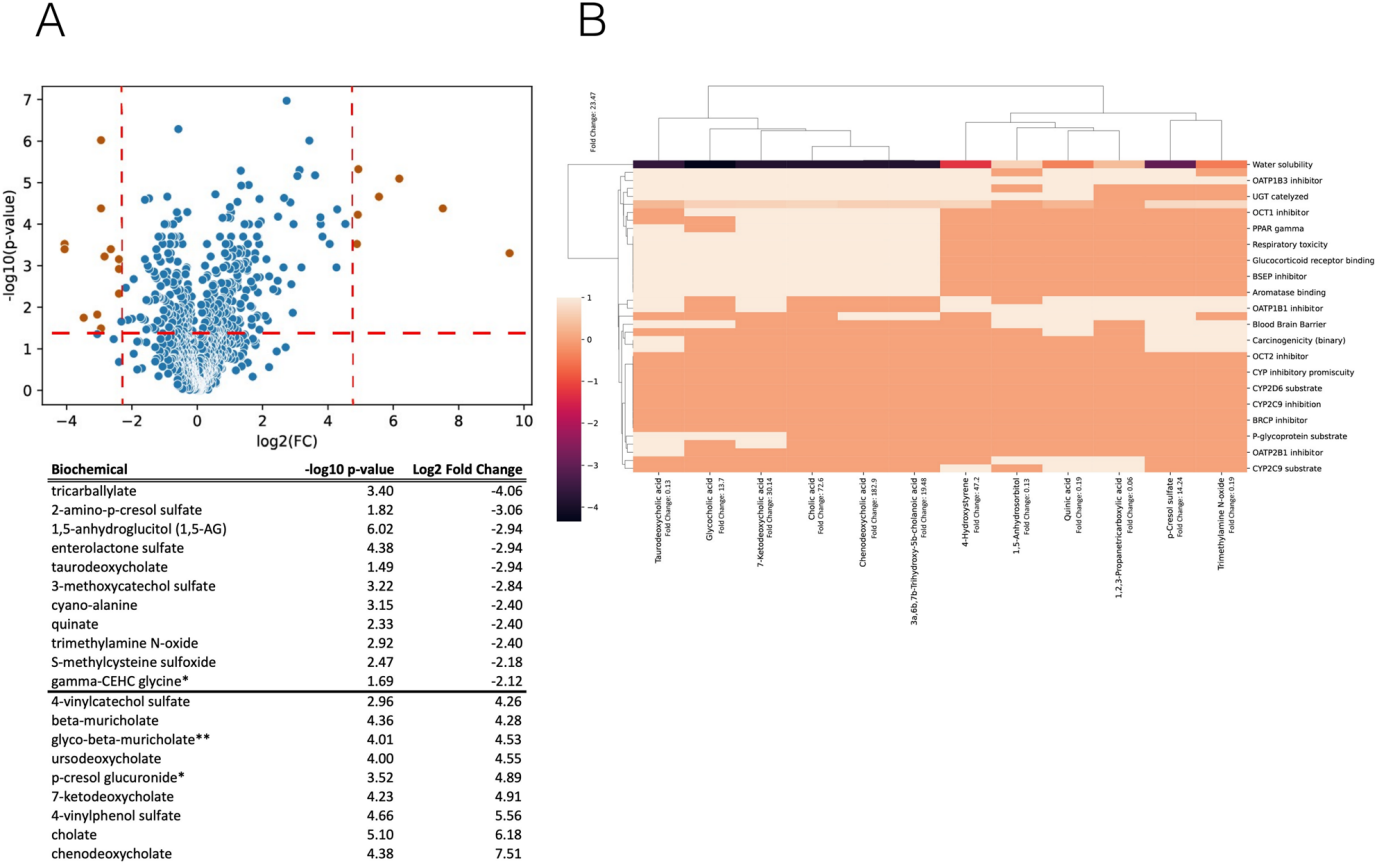
Broad changes across the plasma metabolome. AHR knockout has broad systemic effects, as reflected by the large and broad cohort of plasma metabolome that is altered. This analysis builds on earlier analysis of systemic metabolic changes due to AHR loss^34^. Furthermore, the structural variety of the changed metabolites are not readily explainable by shifts in xenobiotic handling/disposition and simple enzymatic mechanisms. Plasma metabolomics in AHR knockout versus wildtype mice. Summary of quantitative structure–activity relationships for metabolites with the largest and smallest fold changes in plasma concentration in AHR knockout versus wildtype mice. (**A**) Volcano plot of the metabolites. Select outlier metabolites (red labeled) appear in the table below the plot. (**B**) QSAR assessment of significantly (p < 0.05, q < 0.05) increased (> 10 Fold Change) or decreased (< 0.2 Fold Change). There is no clear separation between high (greater than 1) versus low (less than 1) fold change in metabolites and QSAR features.

The plasma metabolome in AHR KO demonstrates broad changes, involving numerous metabolic processes in central and peripheral metabolism. Even after filtering for significantly altered metabolites with several-fold changes, the flagged metabolites span the spectrum of metabolic processes across multiple organs (Supp Table 1). This must be contextualized with our earlier analysis of the AHR knockout metabolome, which focused on the whole body rather that organ crosstalk particularly dependent on renal AHR^32^ (Fig. 2A).

We considered the possibility that quantitative structure–activity relationships (QSAR) could account for these differences. For example, if AHR-dependent reactions were simple enzymatic reactions that are part of a biochemical pathway, then one might expect metabolites of similar structures. However, as observed in Fig. 2B, there was no clear separation between QSAR features and fold-changes or classes/groups of metabolites. The renal metabolome in wild type versus AHR knockout mice also exhibited broad changes in numerous metabolic processes and classes of metabolites (Supp Fig. 1); however, these changes were still distinct from those in the plasma.

While the plasma metabolome shows broad range of effects spanning numerous metabolic functions, it is not clear how these are all functionally related to organ level transcriptomic alterations; a systems analysis is needed. In order to better understand these changes, we sought to explicitly explore the changes in the kidney and the interactions with the liver and microbiome through the addition of plasma metabolomic profiling. Given the extent and the variation between plasma and kidney metabolomes, a more formal, systematic analysis that also accounted for multi-organ interactions was warranted.

### Integrative analysis of the gut microbiome-liver-kidney axis reveals organ-specific alterations

Using kidney transcriptomic, liver transcriptomic, kidney metabolomic, and plasma metabolomic data we reconstructed the gut microbiome-liver-kidney axis (Fig. 1). By mapping these data onto metabolic reconstructions with experimentally validated GPR relationships accounting for tissue specificity (liver and kidney) along with the microbiome, we were able to reconstruct the gut microbiome-liver-kidney axis in silico as a MOMR to evaluate metabolic interactions in wildtype versus AHR knockout in mice. We begin with global metabolic networks (human and representative microbiome) and then layer on the omics data. This approach: 1) culls the content of the network (by removing reactions that are not expressed) and 2) adjusts the feasible flux ranges based on metabolomic changes (Methods). The resulting gut microbiome-liver-kidney axis MOMR can then be explored based on content from individual compartment-specific metabolites and reactions, organelles, and tissue-specific cellular metabolites.

The first level of analysis involved assessing content differences and the capabilities of the different organs based on the omics profiles for the liver and kidney WT versus AHR KO components. So called ‘gain of function’ and ‘loss of function’ as the result of AHR KO can be assessed, similar to our analysis of OAT1 and OAT3 knockouts in mice^33^. Interestingly, although we used metabolomics data from the AHR knockout kidney, we observed a relatively higher number and proportion of altered metabolic reactions in the liver (Fig. 2). This suggests that AHR loss in the kidney may indirectly influence liver metabolism, possibly through inter-organ metabolic regulation.

### Functional, simulation-based analysis of multi-organ metabolic reconstructions reveals alterations in context-specific metabolic pathways

**Fig. 3:**
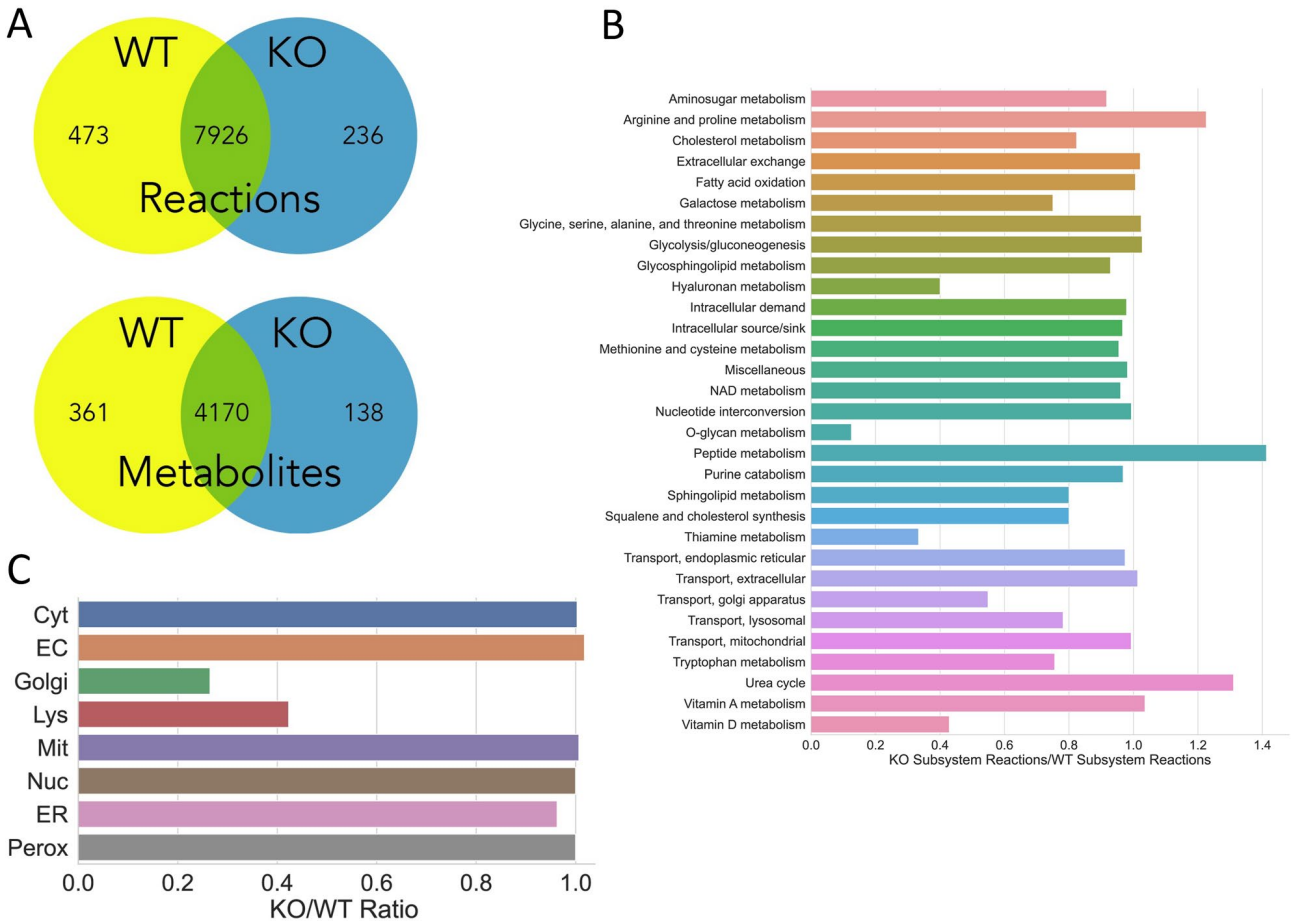

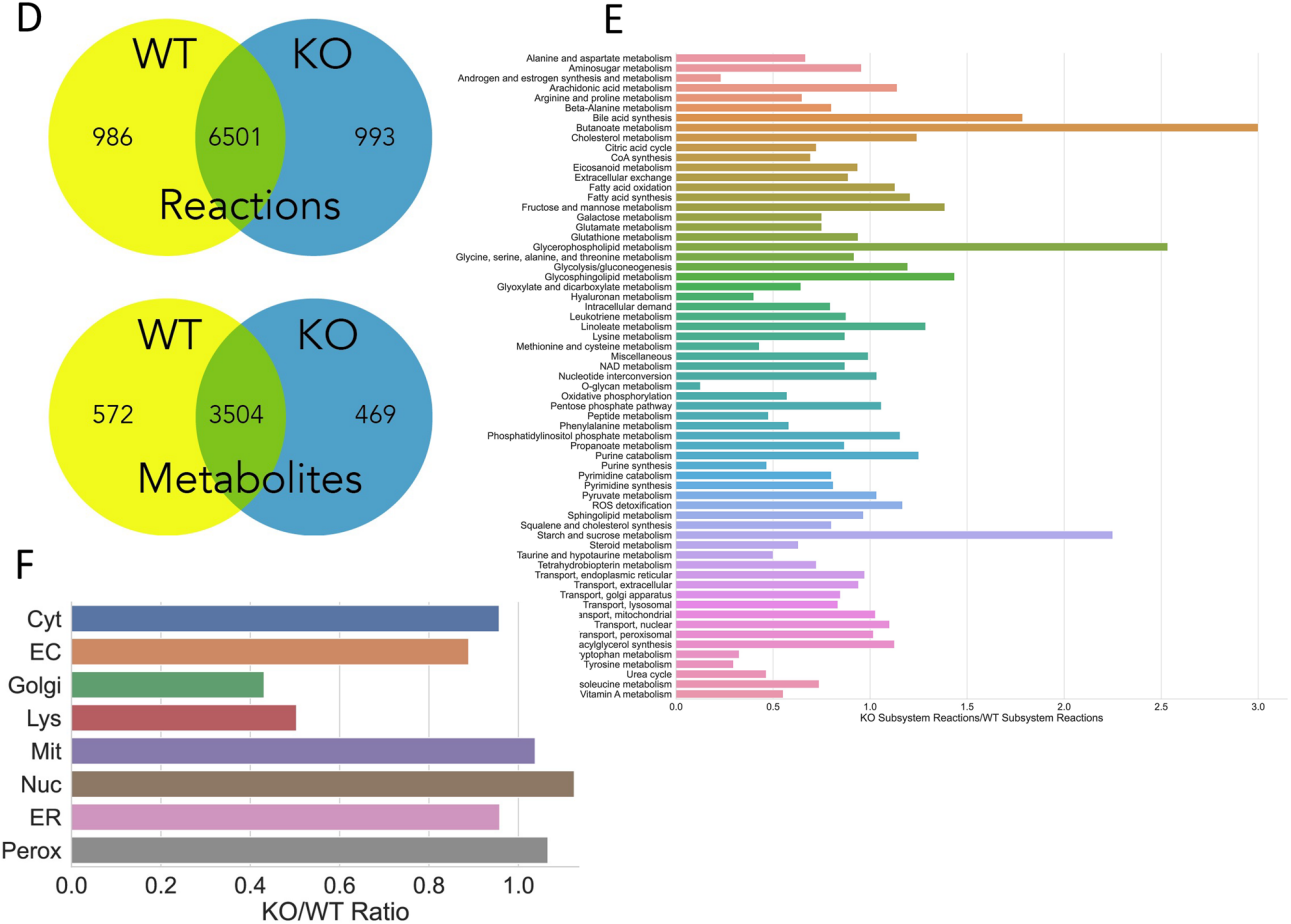
Organ specific reconstruction content comparisons for the AHR KO versus WT reconstruction for the respective isolated kidney (Panels A-C) and liver (Panels D-F) components. (**A**) The isolated kidney component of the Multi-organ Metabolic Reconstructions (MOMR) AHR knockout and wildtype comparison based on network content. Venn Diagrams of the number of compartment-specific metabolites and reactions in the kidney MOMRs. There are approximately twice as many ‘unique’ reactions in the WT as compared to the KO in the kidneys. (**B**) Comparison of content between the two models based on pre-defined network sub-systems. Notable decreases in Hyaluronan and O-glycan metabolism with increases in polyamine related pathways (peptide metabolism and urea cycle). (**C**) Content comparison of metabolomic content according to intra-cellular organelles. Relative decreases in Golgi and lysosomal reactions are observed in the KO models. (**D**) Liver AHR knockout versus wildtype reconstruction content comparison. Venn Diagrams of the number of compartment-specific metabolites and reactions in the liver MOMRs. In contrast to the kidney, there are approximately the same number of unique reactions in the WT and KO Genome-scale metabolic network reconstructions (GEM). (**E**) Comparison of content between the two models based on pre-defined network sub-systems. Notable increases in butanoate, glycerophospholipid, and starch/sucrose metabolism with decreased hepatic tyrosine, tryptophan, taurine/hypotaurine, and androgen/estrogen metabolism. (**F**) Content comparison of metabolomic content according to intra-cellular organelles. Very similar patterns are observed for the liver as the kidney.

When analyzing the models based on simulations, we noted the subset of metabolites unique to the knockout that were increased or related to polyamine metabolism (Table 1). This is consistent with analysis of the isolated kidney and consistent with the literature^34^. Simulations assessing interactions between the different organs and among the different organelles identified more subtle alterations. Notably, these revealed how organelles in different interacting organs were involved in AHR-mediated remote communication. Most notable was a shift in the AHR-dependent interaction between liver ER and liver Golgi apparatus interactions as well as loss of dependencies between kidney nuclear and liver ER metabolites (Fig. 3). Further, in the AHR knockout, there was greater constraints on some of the reactions, suggesting tighter coupling between the kidney and liver.

**Table 1:** Highly connected metabolites that are different in WT versus KO MOMRs.

Metabolites	Compartments
Arginine	kidney-cytosol
CoA and AcCoA	kidney-cytosol
Citrulline	kidney-cytosol
Galactose	kidney-cytosol
Urea	kidney-cytosol
Protons	kidney-mitochondria
Water	kidney-mitochondria
Sodium	kidney-cytosol

**Fig. 4:**
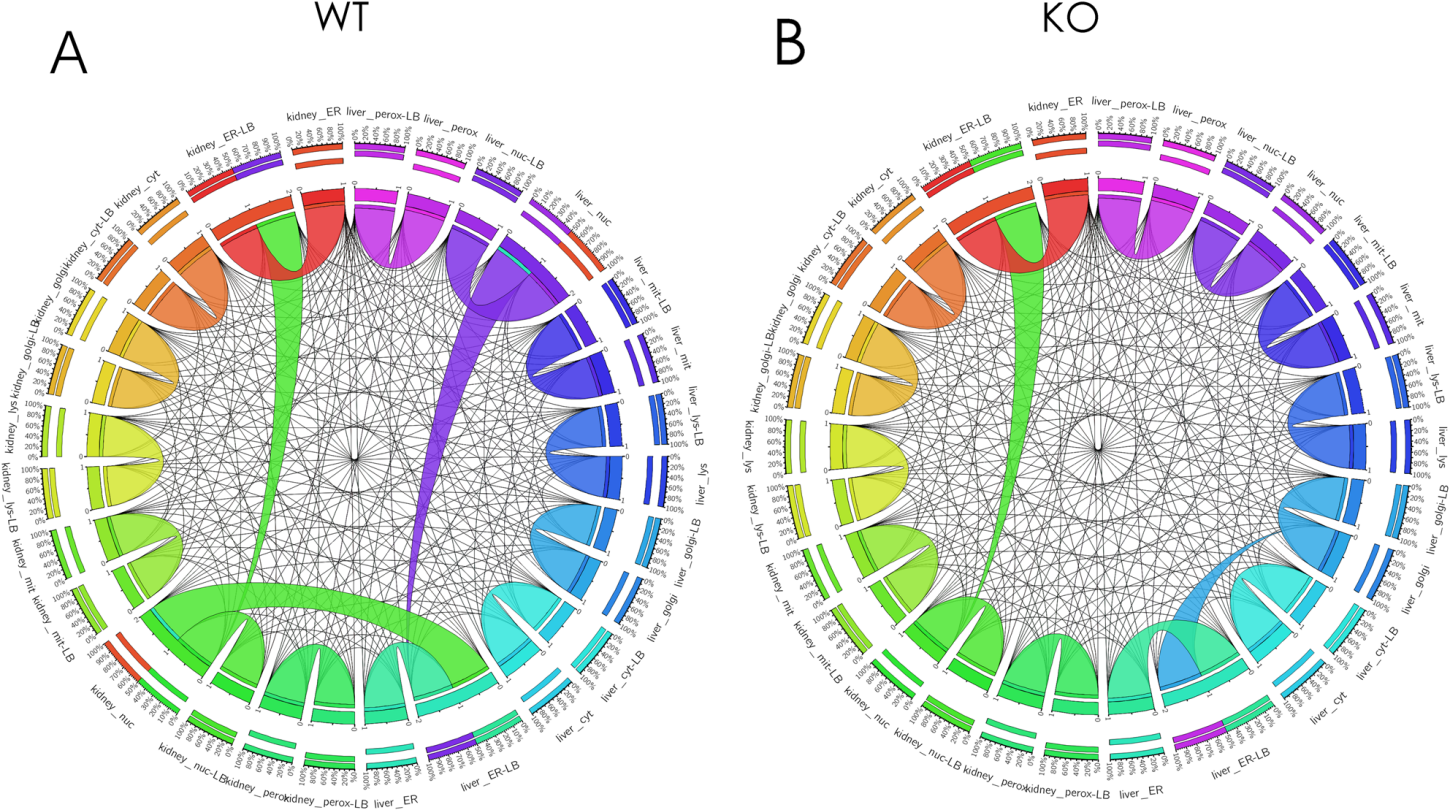
Liver-kidney interactions and metabolic interdependence for AHR WT and KO models. The models were analyzed to assess the influence that different organ metabolic objectives had on one another and whether these changed with the loss of AHR function and visualized with Circos plots. (**A**) Inter-organelle interactions and metabolic interdependence. The Circos plots illustrate direct pairwise interactions and the consequences of a particular organ’s metabolic objective while being directly constrained by a different metabolic demand. The ribbon width of each metabolic reaction to its self-counterpart (i.e. liver to liver-LB) serves as the ‘reference’ comparison for other organ-organ interactions; LB: refers to the condition with a non-zero lower-bound constraint on the tissue-specific demand reaction. Similar ribbon widths reflect relative independence of the corresponding reaction or organ demand. Nuclear and endoplasmic reticulum metabolites were noted to have constraining effects on one another in the liver and kidney, as well as liver and kidney in the wildtype conditions. (**B**) In the AHR knockout, the constraining effect of the endoplasmic reticulum metabolite demand on nuclear metabolites persisted in the kidney. However, there was loss of the constraining effect of the nuclear metabolite demand on the ER and a new constraining influence of the Golgi apparatus metabolites on the endoplasmic reticulum metabolites in the liver. More generally, this illustrates how AHR-dependent remote sensing and signaling between organs involves biochemical reactions in distinct organelles within those communicating organs.

We then proceeded to analyze the context-specific, functional subsystems that were explicitly defined by the transcriptomic and metabolomic integration into MOMR. These analyses showed interdependencies among polyamine and coenzyme A/fatty acids, glycosaminoglycan and ketoacid metabolism, as well as sterol ring and quinone pool metabolism (Fig. 4 and Supp Fig. 3). These processes occur between different organs and organelles in the host and microbiome with quantitative as well as qualitatively altered relationships in the AHR KO as compared to WT. The observed non-linearities observed from the simulations could not have been predicted from the data alone, without the reconstruction. The results also indicated the tighter link between the kidney and liver, as noted from the aforementioned simulations.

**Fig. 5:**
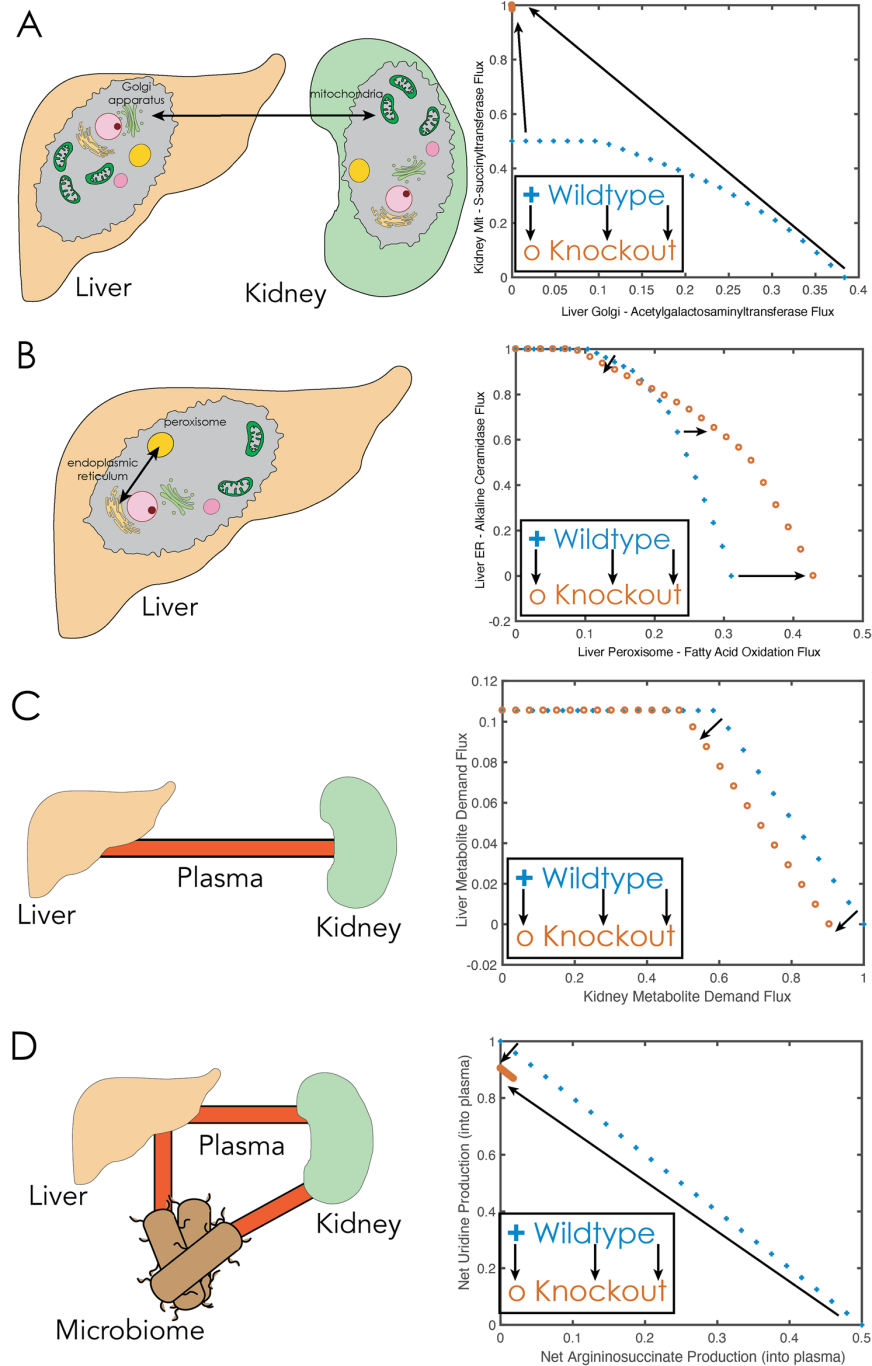
Selected summary of simulation-based comparisons in WT and AHR knockout multi-organ metabolic reconstructions. The robustness plots demonstrate the interdependence of one reaction flux on another, by optimizing one while fixing the other at a non-zero lower bound. Each plotted point represents the optimal state for set of reactions. The black arrows indicate the shifts from the WT to KO condition. Quantitatively and qualitatively altered intra-cellular, inter-cellular, inter-organ, and inter-organism (i.e. host-microbiome) are observed. These examples highlight concretely how the RSST is manifested metabolically. (**A**) The interplay between mitochondrial CoA organic acid metabolism and galactosaminoglycan metabolism in the liver are depicted showing much tighter coupling and constraint between the two processes in the AHR KO. Model reaction abbreviations for the ordinate and abscissa are r0557_kdny and GALNACT2g_lvr, respectively. We see that the antagonistic relationships between the two reactions becomes compressed down to a very narrow range (effectively a single point), reflecting decreased flexibility between CoA metabolism and hepatic galactosaminoglycan metabolism. (**B**) Ceramidase metabolism versus peroxisomal fatty acid metabolism in the liver shows a shift towards the latter in AHR KO. The specific reactions for the ordinate and abscissa are ACER11r_lvr and FAOXC121C10x_lvr, respectively. All axes are normalized to the maximum of the ordinate and abscissa vectors for each plot, respectively. (**C**) The liver metabolome demands versus kidney metabolome demands remain qualitatively similar, but quantitatively different in WT versus AHR KO. Above a relative flux of 0.5 for the kidney and ~ 0.1 for the liver, the biochemical organ demands are reciprocally related at a fixed stoichiometric ratio and then become independent of one another below those levels. (**D**) The trade-off between uridine production into the plasma compartment versus argininosuccinate which on average is increased in the knockout condition, but also significantly compressed into a narrower range in AHR KO. This suggests tighter linking between these metabolites in AHR KO. Reaction formulas and abbreviations: r0557_kdny: Glutaryl-CoA:dihydrolipoamide S-succinyltransferase (coa_m + HC01712_m ⇌ dhlam_m + glutcoa_m), GALNACT2g_lvr: Uridine diphosphoacetylgalactosamine-chondroitin acetylgalactosaminyltransferase II, Golgi (cs_a_b_pre2_g + uacgam_g ⇌ h_g + cs_a_b_pre3_g + udp_g). ACER11r_lvr: Alkaline Ceramidase 1/Alkaline Ceramidase 3 (h2o_r + crm_hs_r ⇌ sphings_r + Rtotal_r), FAOXC121C10x_lvr: Fatty Acid Beta Oxidation (C12:1- > C10), Peroxisomal (coa_x + dd2coa_x + h2o_x + nad_x ⇌ accoa_x + dcacoa_x + h_x + nadh_x), HC01712: S-Glutaryldihydrolipoamide, Dhlam: Dihydrolipoamide, glutcoa: Glutaryl-CoA, cs_a_b_pre2_g: Chondroitin sulfate A (GalNAc4S-GlcA) and B (IdoA2S-GalNAc4S), precursor 2, cs_a_b_pre3_g: Chondroitin sulfate A (GalNAc4S-GlcA) and B (IdoA2S-GalNAc4S), precursor 3, uacgam_g: UDP-N-acetyl-D-glucosamine, udp_g: UDP, crm_hs_r: Ceramide, sphings_r: Sphingosine, Rtotal_c: Hydrocarbon chain, accoa_x: Acetyl-CoA, coa_x: Coenzyme A, dcacoa_x: Decanoyl-CoA (n-C10:0CoA), dd2coa_x: Trans-Dodec-2-enoyl-CoA, nad_x: Nicotinamide adenine dinucleotide, nadh_x: Nicotinamide adenine dinucleotide (reduced).

Simulation based studies can also illuminate quantitative dependencies in metabolism and identify qualitative alterations in AHR KO. Figure 5 highlights interactions, explicitly showing how the Remote Sensing and Signaling Theory manifests in inter-organ (Fig. 5A and 5C), intracellular (Fig. 5B), and multi-organ and microbiome (Fig. 5D) with alterations in the AHR KO mice. Many relationships and dependencies in metabolism follow “supply and demand” type interactions in different cells or organs compete for limited resources. Thus, relationships between two “competing” organs have anti-correlated relationships. One can the identify that, in some regions (low metabolic demand), two organs may be independent of one another. But at higher demand conditions, a competing limitation is observed (e.g. Figure 5C). Furthermore, in some cases (a linear anti-correlated relationship is collapsed to a narrow, tightly constrained range in the AHR KO, as see with argininosuccinate versus uridine production (Fig. 5D). Additionally, one can identify how functional, flux-based subsystems change in the WT versus KO condition (Supp Fig. 3, Supp Fig. 4, Tables 2 and 3). While the majority of the subsystems are stable, a number of them were either lost, or gained as a result of HR knockout.

**Table 2:** WT MOMR co-sets that were “lost” in AHR KO.

WT Co-sets (“Lost” in AHR KO)	CoSetSize/Pathway length
Kidney-cholesterol	14
Kidney-cholesterol 3	7
Kidney-chondroitin 4	8
Kidney-Ethanolamine, Choline, Adenosylmethionine	12
Kidney-Fatty acid	12
Kidney-Fatty acid 5	9
Kidney-Fatty acid 6	9
Kidney-Fatty acid 7	8
Kidney-Fatty acid 8	8
Kidney-Fatty acid 9	7
Kidney-Glycosphingolipid	8
Kidney-Glycosphingolipid 4	7
Kidney-Glycosphingolipid 5	7
Kidney-Glycosphingolipid 6	7
Kidney-Heparan sulfate	48
Kidney-Keratan sulfate	14
Kidney-Keto-Aldehyde-Redox	7
Kidney-Leukotriene	7
Liver-Carnitine 2	7
Liver-Cholesterol	14
Liver-Chondroitin	13
Liver-Chondroitin 2	10
Liver-CoA/Carnitine 2	6
Liver-Fatty acid oxidation, mitochondria 5	7
Liver-IMP	8
Liver-Keratan sulfate	14
Liver-Leukotriene	7
Liver-Microbiome	9
Microbiome-Branched AA degradation 2	6
Microbiome-Glucosamine	6
Microbiome-IMP	7
Microbiome-Sugar	16

There are 32 co-sets that are present in the WT MOMR but are lost/disrupted in the KO; 18 involve the kidney (average pathway length of 11 reactions), 10 involve the liver (average pathway length of 9.5 reactions), and 4 involve the microbiome (average pathway length of 9 reactions).

**Table 3:** KO MOMR co-sets that were “gained” in AHR WT.

KO Co-Sets (“Gained” in AHR KO)	CoSetSize/Pathway length
Kidney-Carnitine	8
Kidney-Carnitine 2	6
Liver-Chondroitin 5	7
Liver-Chondroitin/Heparan sulfate	19
Liver-Fatty acid 3	10
Liver-Fatty acid 4	6
Liver-Fatty acid oxidation, Mitochondria 8	11
Liver-Fatty acid oxidation, Peroxiosme 7	6
Liver-Fatty acid oxidation, Peroxisome 10	6
Liver-Fatty acid oxidation, Peroxisome 11	6
Liver-Fatty acid oxidation, Peroxisome 8	6
Liver-Fatty acid oxidation, Peroxisome 9	6
Liver-Fatty acid oxidation, Peroxisome-KO	12
Liver-Fatty acid transport	7
Liver-Kidney amino acid	6
Liver-Kidney amino acid 2	6
Liver-Kidney amino acid 3	6
Liver-Kidney carnitine/CoA	8
Liver-Kidney fatty acid	8
Liver-Kidney fatty acid 2	8
Liver-Sphingolipid	8
Liver-Sphingolipid 2	6
Liver-Sphingolipid 3	6
Liver-Sphingolipid transport	7
Liver-Sterol	10
Liver-Transport	9
Microbiome Folate	11
Microbiome-Leucine	6
Microbiome-Sugar 2	6
Microbiome-Sugar 3	6
Microbiome-Transport	7

There are 31 co-sets that appeared/developed with AHR loss; 2 sets involve the kidney (average pathway length of 7 reactions), 18 sets involve the liver (average pathway length of 8 reactions), and 5 sets involve the microbiome (average pathway length of 7 reactions). Notably (and unique to the “gain” of co-sets), there are 6 co-sets that involve liver-kidney reactions (average pathway length of 7 reactions).

### Assessment of discordant metabolites informs new potential cofactor-dependent nitrogen handling pathways

**Fig. 6:**
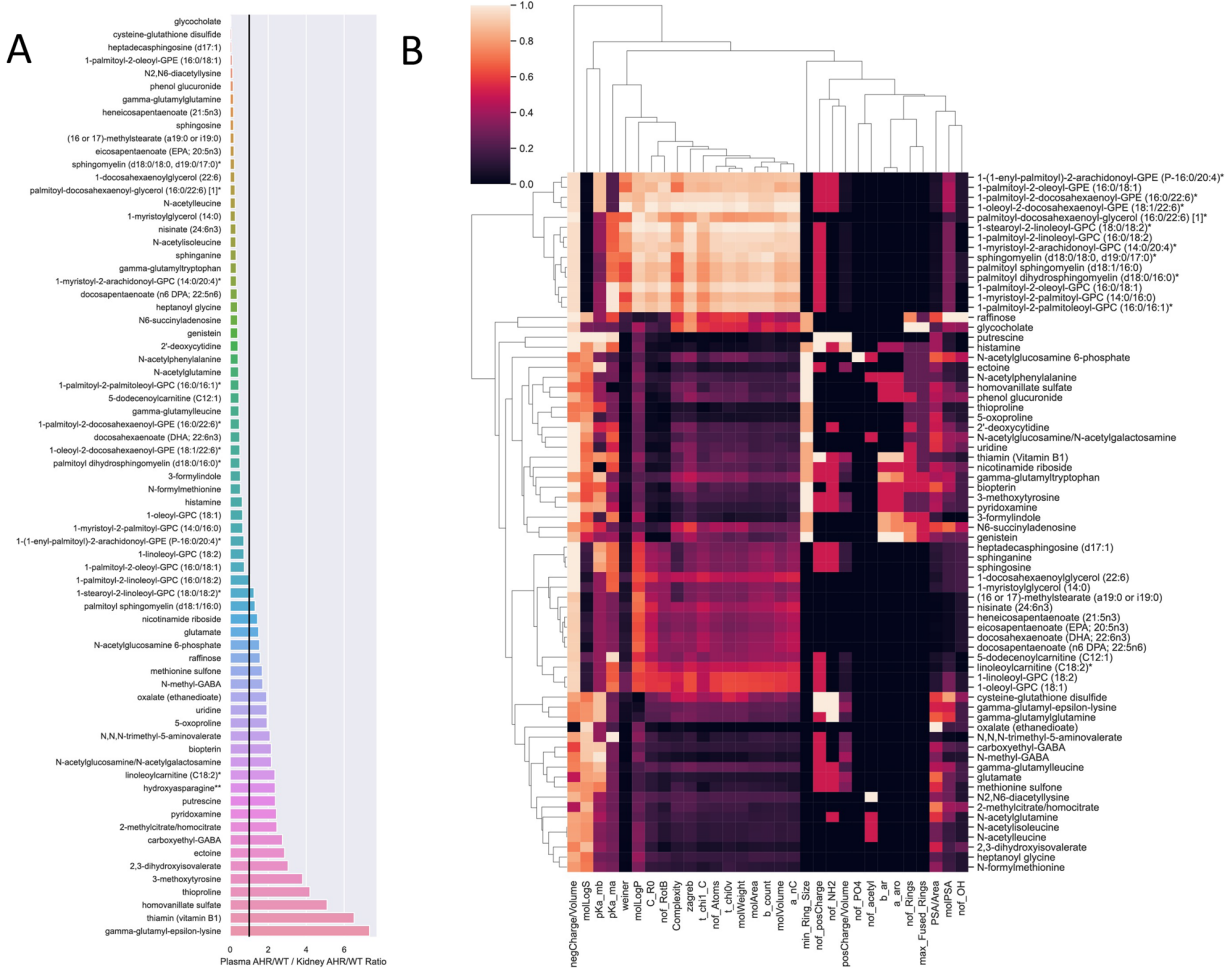
Discordant metabolites identify reaction pathways that are disrupted in kidneys in AHR KO mice. We classified the significantly altered the kidney metabolome into two groups, concordant metabolites for which the KO/WT ratio for plasma and IC kidney are both either greater than 1 or less than 1, in contrast to discordant metabolites for which the KO/WT ratio for plasma and IC kidney are NOT in agreement. We focus on discordant metabolites, as these likely reflect actively regulated processes. (**A**) Bar chart of discordant metabolites. Metabolite fold changes that are either less than one or greater than one appear to the left versus right side of the black line, respectively. (**B**) Clustered heat map of chemoinformatic properties of the discordant metabolites. There are two main branches of the clusters based on the chemical properties, driven by properties such as molecular weight, structure complexity, molecular area, and molecular volume versus number of functional groups (e.g. phosphate, acetyl, ammonium, hydroxyl), number of rings, and charge. Consequently, we see separation of metabolites (y-axis) along groups based on biochemical moieties, such as acetylation, free fatty acids, and conjugated fatty acids.

The severely constrained relationships between argininosuccinate versus uridine production in the AHR KO as compared to the WT MOMR (Fig. 5D) identified nitrogen handling as a potentially consequential metabolic alteration in loss of AHR expression. This led us to further explore the metabolomics and qualitative changes in gene expression in the context of the nitrogen handling pathways. We classified metabolites in terms of discordant versus concordant metabolites based on whether the fold-change ratios of KO to WT metabolite concentrations were consistent between the kidney cells and the plasma compartment. Concordant metabolites show multi-fold changes in the same direction for the intracellular kidney metabolites and the plasma, which might be explained by simple concentration gradient-based transport across cells and membranes. Discordant metabolites, those in which the directions of full changes were in opposing directions, likely involve some type of actively regulated translational, post-translational, or transport process (Fig. 6).

Many of the indole sulfates (indolepropionate and indolepropionylglycine) were concordant between the intracellular kidney and plasma metabolomics. 3-formylindole (indole-3-carboxaldehyde), also a microbial derivative of tryptophan, was an exception to the general trend and was thus a discordant metabolite. Interestingly, 3-formylindole is a known AHR agonist, which may provide a partial explanation for the non-mass action associated activity (i.e. different renal versus plasma changes in WT versus KO)^35,36^.

**Fig. 7:**
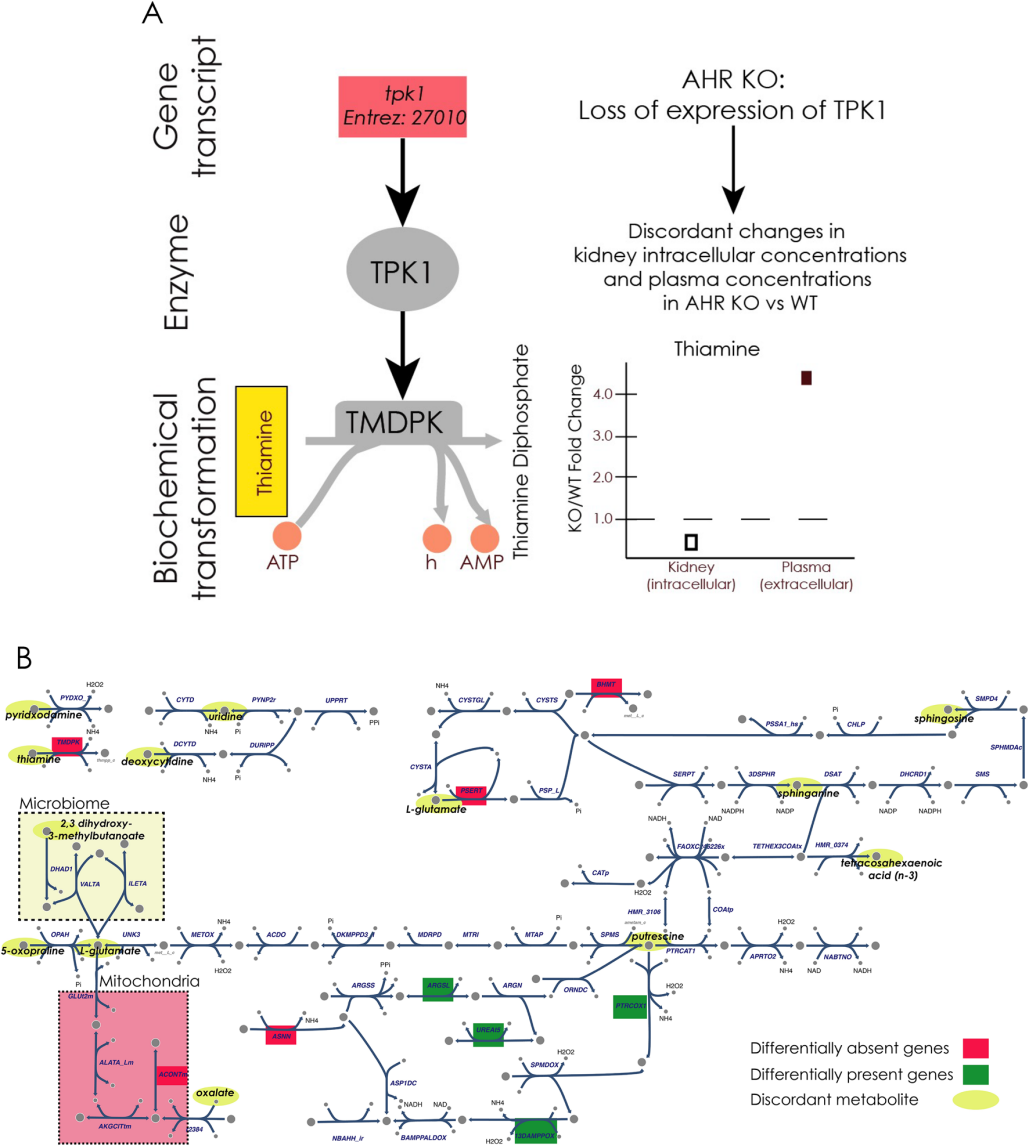
Concordant versus discordant metabolite changes on a network level. (**A**) As an illustrative example, the GPR relationship for thiamine pyrophosphokinase 1 (TPK1) is depicted. AHR KO exhibited loss of expression of *tpk1*. Based on this, one would expect decreased maximal flux from the corresponding phosphoserine transaminase (PSERT) reaction. However, the metabolomic data demonstrated discordant changes in the intracellular glutamate concentration as compared to the plasma concentration. This suggests that strict mass action in the context of the biological relationships cannot account for the observed data and actively regulated process(es) is(are) due to AHR KO are maintaining the change in the glutamate concentration changes in the different compartments. (**B**) Network map of metabolism involving amino acid (particularly nitrogen handling metabolic reactions) and fatty acid pathways, highlighting specific discordant transcriptome-metabolome alterations from AHR loss in the kidney. Oval yellow nodes correspond to discordant metabolites. Red and green squares highlight the enzymes (and corresponding genes) that are differentially absent or present in the AHR KO compared to WT. Disruption of AHR expression results in changes in polyamine related pathways that are coupled to redox cofactor balances, ammonia handling, and superoxide production. Previous work looking at AHR KO in liver and MCF7 cells identified functional pathways that were altered with respect to nitrogen handling^34^. The map was updated around the discordant metabolites as well as genes that were differentially present/absent in AHR KO vs WT. The pathways focus around the phosphate-dependent and NAD-dependent pathways for nitrogen moiety metabolism with the addition of intracellular kidney metabolomic data. The changes in enzyme expression of key reactions in the vicinity of these metabolites indicate potential regulatory roles for the corresponding genes. Thus, this map provides a draft outline for a regulatory hub to account for regulation of nitrogen handling alterations in response to AHR loss. Metabolite abbreviations: acile__L_c: acetyl isoleucine, acleu__L_c: N-acetyl-leucine, gchola_c: glycocholate, Pi: orthophosphate, PPi: pyrophosphate, h2o2: hydrogen peroxide. Abbreviations for metabolites and reactions use nomenclature from the BiGG database (http://bigg.ucsd.edu/).

Focusing on the subset of discordant metabolites that mapped to the model, we evaluated these in the context of the nitrogen handling pathways that were recently described^34^. On top of this, we layered the enzymes that were differentially present or absent in the AHR knockout (Fig. 7A). In particular we note that: 1) the discordant metabolites involve metabolites involved in a structurally and functionally wide range of biochemical species (fatty acids, organic acids, polyamines, amino acids, and nucleotides); 2) the set of metabolites are connected through metabolites of ammonium, phosphate, redox cofactors, and hydrogen peroxide metabolism; 3) up-regulation/increased expression of urea cycle/nitrogen handling enzymes with down-regulation of the enzymes that are more distant or not directly involved in nitrogen handling. This network map shows how broad metabolic processes and metabolites (Supp Fig. 1 and Fig. 7) are physically linked together by biochemical processes that are increased or decreased as reflected through shifts in enzyme availability (Fig. 7B). Further investigation is needed to determine the mechanisms underlying this systemic effect.

## Discussion

It is worth emphasizing the crucial importance of the kidney in drug and toxin excretion, usually through the action of multi-specific SLC and ABC transporters^19,37,38^. As a ligand-activated receptor, AHR is well known to bind drugs and toxins as well as regulate many of the SLC and ABC transporters involved in drug and toxin handling in the kidney–and other organs such as the liver. However, the emphasis here is on the endogenous metabolic role of AHR in the kidney, as well as its role in remote communication along the gut microbiome-liver-kidney axis. Our results provide a better basis for understanding the organ crosstalk as well as non-obvious metabolic effects of drugs and toxins that enter the kidney and bind AHR.

AHR has also been invoked as an example of a proximal tubule sensor in the Remote Sensing and Signaling Theory (RSST)^12,20,31,39–41^. Several labs have now shown remote sensing and signaling in the normal kidney and in 5/6th nephrectomy models of CKD^28,29,31,32,42^. One of the less well-studied aspects of the remote sensing and signaling mechanism in the kidney is the particular metabolic function of renal AHR. Our results provide the first multi-omics analysis of kidney AHR dependent reactions in the kidney as well as the liver and gut microbiome. This novel Multi-Organ Metabolic Reconstruction (MOMR) should be of broad interest in metabolism, pharmacokinetics, toxicology, and uremic toxin biology.

AHR is expressed in multiple organs and there is considerable complexity in these interactions and pathways across multiple scales. Because of this, conventional classification and analysis methods are not sufficiently sensitive or specific to detect more subtle changes in inter-organ communication or intracellular alterations. The MOMR presented here is a multi-level systems approach that incorporates the principles of detailed balance of small molecule metabolism and transport, along with intracellular compartmentation, multi-organ interactions, and microbiome metabolism (Fig. 3). It is a highly novel approach to defining how metabolism across organs and organisms (gut microbiome) is altered due to AHR loss in the kidney.

Through a data-driven, multi-organ constraint-based analysis of kidney and liver transcriptomics along with kidney intracellular metabolomics, and plasma metabolomics, we were able to find: 1) metabolic alterations affecting the renal modulation of fatty acids, organic acids, amino acids, and nucleotides; 2) a set of metabolites central to the oxidative stress response in the kidney, including redox cofactors, and hydrogen peroxide metabolism; 3) alterations in urea cycle enzymes, polyamines, and enzymes of involved in nitrogen handling. The results of the MOMR analyses provide a concrete manifestation of how remote sensing and signaling integrated by a master transcription factor (AHR) can operate biochemically across a spatial hierarchy spanning sub-cellular organelles to host-microbiome interactions (e.g. Figures 4 and 5). These concepts have been extensively discussed in the experimental and other literature related to the Remote Sensing and Signaling Theory, which focuses on remote inter-organ and inter-organismal communication via transported small molecules^12,18–20,24,31–36,47^.

Some of the implied physiological functions, such as the handling of organic acids and nitrogen are key functions of the kidney. Regulation of redox state appears to be a very important role of the proximal tubule in health and acute injury as well as in mouse models of diabetic renal injury^43,44^. AHR deficiency attenuates oxidative stress-related mesangial cell activation and macrophage infiltration and extracellular matrix accumulation in diabetic nephropathy^43^. Polyamines and other metabolites play a key role in cell proliferation and fibrosis^45–49^. Although the specific mechanisms underlying renal fibrosis are only partly understood, these proteins and metabolites are found in many mechanistic discussions^50,51^, and AHR activation after uptake of indoxyl sulfate into the proximal tubule is associated with TGF-β production^52^. Indoxyl sulfate downregulates expression of Mas receptor via OAT3/AhR/Stat3 pathway in proximal tubular cells^52^, which is known to play a role in fibrosis. Our results are generally consistent with the view that drugs targeting AHR might be a fruitful avenue for counteracting renal injury and/or fibrosis.

Numerous AHR ligands are tryptophan derivatives that are synthesized or modified in the gut microbiome. These include indole-derivatives such as indoxyl sulfate and indole acetic acid. Other tryptophan derivatives that bind AHR arise through the kynurenine pathway. Some of these are among the key “uremic toxins” of CKD^31^, resulting in progressive kidney and cardiovascular disease through their activity within the gut-heart-kidney axis^53^.

This pathway in the kidney has attracted a great deal of attention because its activation is thought to result in renal fibrosis and worsening kidney function^54,55^. Temporal and tissue-specific activation of AHR occurs in discrete mouse models of kidney disease^56^. The transport of indoxyl sulfate via the organic anion transporters, OAT1 (SLC22A6) and OAT3 (SLC22A8), results in uptake of indoxyl sulfate into proximal tubule cells, whereupon AHR activation occurs^20,39^. As we have shown here, AHR activation in the kidney results in a broad range of metabolic alterations along the gut microbiome-liver-kidney axis, some of which are common to systemic AHR activation^34^.

### Study limitations

There are multiple study limitations that must be acknowledged. It is well recognized that sex is a biological variable and metabolism differences would be expected in multi-organ models. The metabolomic data was generated from mice with different genders. Since the same mix of female and male mice were used in each of the respective analyses, this is a source of potential systematic error. We would expect such errors to arise in the detection or analysis of sex-specific steroid compounds. Since our results and analyses did not focus on these metabolites, we believe that the likelihood of such errors are low. Additionally the MOMR builds upon FBA based approaches to include multi-organ multiomics, however these models do not account for many important biological processes. This is a steady state/non-kinetic model, so further nuances related to altered kinetics are not captured in the current scope of this model. However, this provides an initial roadmap that may help guide the future development of dynamic and pharmacokinetic models.

We have observed that drug transporters that modulate exogenous as well as endogenous metabolic processes involve the synthesis and degradation of metabolites with wide-ranging chemical structures and chemical moieties. Importantly, the biological process at hand does not involve canonical pathways, but rather involves numerous pathways that converge around cofactor balances and maintaining redox states. While principles of redox and energy balance have been demonstrated to be of importance in simpler (enucleate) cells^57–60^, studies explicitly accounting for the intracellular transformations along with inter-organ interactions (e.g. gut microbiome-liver-kidney axis) have been lacking. It is recognized that maintaining redox states through cofactor balances and energy states is essential for core biochemical functions that must be present for higher level transcription and gene regulatory processes to operate^61–64^. Through systematic analysis of data-driven reconstruction of the gut microbiome-liver-kidney axis, it is possible to appreciate how a master transcriptional regulator in one organ (kidney) regulates systemic metabolism based on its effects on multi-organ correlated biochemical reaction sets–in essence, organ cross-talk mediated through transported small molecules.

## Methods

### Animals

As described previously by Granados et al^34^, C57B6.129-Ahrtm1Bra/J (Ahr−/−) mice samples were from Dr. Gary Perdew (The Pennsylvania State University). The mice maintained with *ad libitum* access to water and standard rodent chow in a pathogen-free vivarium. 8–10-week-old female Ahr −/− (n = 7) and wildtype mice (n = 5) were euthanized by carbon dioxide asphyxiation (1L/min) (Supp Table 1).

Previous validation studies demonstrated that n ≥ 4 provides sufficient power to identify robust metabolite shifts while limiting false positives^65^. The number of Ahr -/- mice are nearly twice the number of wildtype in order to minimize variance and improve the signal to noise ratio for the group-wise comparisons. Tissue and sera samples were stored at − 80 °C until analysis. The study was approved by Pennsylvania State University Institutional Animal Care and Use Committee, Protocol Number: 201901049. All experiments were carried out in compliance with ARRIVE guidelines.

### Metabolomics data analysis

Serum and bulk intracellular kidney samples were sent to Metabolon (Durham, NC) for metabolomics targeting profiling. Intensity values were normalized to volume, log transformed, and missing values were imputed with the lowest measured value for each metabolite. Welch’s t-test was used to determine statistical significance between groups, and false discovery rate (FDR)-adjusted p-values and q-values were calculated. A simpler analysis of the plasma metabolomics (but not the kidney) has been previously described and was used to contextualize the metabolic reconstructions performed here^34^.

### Transcriptomic data analysis

Transcriptomic datasets for AHR activation/inhibition were acquired from the Gene Expression Omnibus (GEO) database (www.ncbi.nlm.nih.gov/geo/, Boutros et al.^66^, GEO Accession: GSE15859) and were analyzed for differentially expressed genes using Bowtie, SAMtools, bioMart, and DESeq in R (R v 4.2.2). These samples correspond to the liver and kidney tissue obtained from male Ahr -/- mice and their wild-type counterparts, male C57BL/6 mice, from The Jackson Laboratory (Bar Harbor, ME), as described by Boutros et al.^66^.

### Metabolic reconstructions

Our systems approach to interpreting the multi-omics datasets involved using metabolic network reconstructions and constraint-based modeling. Host-microbe integration^67^ of Recon3D^25^ and *Bacillus subtilis* (iYO844)^68^ metabolic network reconstructions served as the base for the subsequent context-specific reconstruction. Since AHR is generally expressed in multiple tissues, and since plasma metabolomics reflect interactions with multiple tissues and organs, we used the reconstruction as context to interpret changes in gene expression and plasma/kidney metabolomics. Since reaction fluxes do not correlate with gene expression quantitatively, in general, we used present/absent calls for biological relevance. Thus, we used the given present/absent calls to interpret the up and down regulated genes of interest by comparing WT and AHR knockout calls. Since AHR is expressed in a multitude of tissues, we did not target a particular organ with the transcriptomics data but rather the systemic consequences of the altered expression. The results provide a view of the functional metabolic pathway consequences resulting from differential activity of the reactions corresponding to these genes. Simultaneously, the metabolomic data changes in kidney and plasma were incorporated through adjusting the corresponding exchange reaction constraints.

In order to minimize non-specific calculations, only measured metabolites (in addition to ions, water, and oxygen) were available for cellular uptake by the models. Flux Variability Analysis (FVA) was used to identify the subset of metabolites that were strictly taken up or strictly secreted (metabolites that can be taken up and/or secreted cannot be used to directly constrain the model, because the circulating plasma compartment communicates with other organs in the body that may in turn uptake or secrete any particular metabolite). The resulting calculations based upon metabolomic data constraints were used to classify metabolites into three groups, 1) those that can only be produced, 2) those that can only be taken up, and 3) those that can be secreted or taken up. Metabolomic fold changes (KO relative to WT) were implemented as constraints on the models using the following conditional statements:

- KO/WT > 1 and the metabolite could only be produced by the model, then the metabolite exchange was constrained with a lower bound set to 50% of the maximum production
- KO/WT > 1 and the metabolite could only be taken up, then the metabolite exchange was constrained with a lower bound that was 50% of the minimum uptake
- KO/WT < 1 and the metabolite could only be produced by the model, then the metabolite exchange was constrained with an upper bound that was 50% of the maximum production
- KO/WT < 1 and the metabolite could only be taken up, then the metabolite exchange was constrained with upper bound that was 50% of the minimum uptake

The GiMME algorithm^69^ was used to construct wildtype and *AHR* knockout models using transcriptome present/absent calls. Similar to Jamshidi and Nigam^33^, since direct flux measurements were not available and the targeted analysis was to assess differences in the WT vs KO conditions, an arbitrary reference uptake of 0.25 mM/gDW/hour was specified for the reference WT models and the metabolomic constraints for the set of metabolites that were significantly altered in the WT and KO were applied. As before, ‘free’ uptake was permitted for oxygen, sodium, potassium, iron, magnesium, bicarbonate, protons, and water.

#### Integration of models

Once each tissue specific model was constructed (wildtype and knockout for the liver and kidney), the models were merged according to the previously described protocol^67^, with the modification to merge in the extracellular (plasma) compartment instead of intracellular. Metabolites and reactions were post-fixed with abbreviations “lvr” for liver, “kdny” for kidney, and “bsc” for the representative microbiome. Post-fixed, lower case abbreviations specifying extra- and intra-cellular locations include, c: cytosol, e: extracellular, g: Golgi Apparatus, l: lysosome, m: mitochondria, n: nucleus, r: endoplasmic reticulum, and p: peroxisome. Subsequent flux balance analysis simulations were performed for the merged MOMR models (kidney-liver-microbiome WT and KO). Differential mean flux states were then computed from the normalized sampled feasible flux states, requiring the following conditions: p < 0.001 for the Kolmogorov–Smirnov test as well as the F-test, with either greater than fivefold change or less than 0.2 fold change as described previously^33^. Tissue metabolic demand reactions were defined for the liver and kidney, individually, as $\sum_{i=1}^{m}{x}_{i}^{*}{x}_{i}$ where ${x}_{i}^{*}$ is the maximum MOMR net production of metabolite, ${x}_{i}$.

### Circos plots

Circos plots^70^ were generated following calculation of pairwise interactions between competing organ metabolic objectives, similar to a previously described approach^44^. The purpose was to identify functional metabolic dependencies with wildtype mice as well as AHR knockout mice to see how systemic metabolic states are affected by loss of AHR function. Comparisons were made following calculation of a matrix, M, whose entries m_ij_ are defined as max(c_j_) for i = j, and max(c_i_) for i $\ne$ j, with i, j $\in {N}_{+}^{n}$ , and v_j_^l^ = α*max(c_j_) for j subset of the reaction list. For each vector, a set is calculated covering a range of α ∈ (0,1], using with objective vector, c ∈ {0,1}^n^ and $\sum_{i=1}^{n}{c}_{i}=1$. Interpretation of Circos plots: Each demand or pseudoreaction is compared to a reference reaction that has been set to 0.99 times the maximum of that reaction. This is similar to the Circos plots described in^44^, except the lower bound is set to approximately the maximum flux. The purpose of this calculation is to highlight potential differences between the wildtype and knockout conditions.

Model modifications and simulations were carried out with using CobraPy and the CobraToolbox with the Gurobi Optimizer (v 8.0)^71,72^. The network pathway map was created using Escher (escher.github.io)^73^. Circos plots were generated with Circos version 0.69-6^70^. Plots and figures were generated using Python (v 3.7.1, python.org) and Adobe Illustrator CC 2015 (Adobe Inc, San Jose, CA).

## Supplementary Information

Supplementary Information 1.

Supplementary Information 2.

Supplementary Information 3.

## Data Availability

All data generated or analyzed during this study are included in this published article, its supplementary information files, and cited references. Further questions can be addressed to the corresponding author, N.J. at njamshidi@mednet.ucla.edu.

## Abbreviations

AHR: Aryl hydrocarbon receptor
ADME: (Drug) Absorption, distribution, metabolism and excretion
ANOVA: Analysis of variance
CKD: Chronic kidney disease
COBRA: Constraint-based reconstruction analysis
DME: Drug metabolizing enzymes
FBA: Flux balance analysis
FVA: Flux variability analysis
GEM: Genome-scale metabolic network reconstructions
GPR: Gene-protein-reaction (relationship)
KO: Knockout
MOMR: Multi-organ metabolic reconstructions
QSAR: Quantitative structure–activity relationships
QSP: Quantitative systems pharmacology
RSST: Remote sensing and signaling theory
TSEA: Tissue specific expression analysis
WT: Wildtype
Post-fix: _Kdny: the kidney compartment
Post-fix: _Lvr: the liver compartment

## References

[1] Barouki, R., Aggerbeck, M., Aggerbeck, L. & Coumoul, X. The aryl hydrocarbon receptor system. *Drug Metabol. Drug Interact.* **27**(1), 3–8. 10.1515/dmdi-2011-0035 (2012). 22718620 10.1515/dmdi-2011-0035

[2] Rothhammer, V. & Quintana, F. J. The aryl hydrocarbon receptor: An environmental sensor integrating immune responses in health and disease. *Nat. Rev. Immunol.* **19**(3), 184–197. 10.1038/s41577-019-0125-8 (2019). 30718831 10.1038/s41577-019-0125-8

[3] Stockinger, B., Di Meglio, P., Gialitakis, M. & Duarte, J. H. The aryl hydrocarbon receptor: Multitasking in the immune system. *Annu. Rev. Immunol.* **32**, 403–432. 10.1146/annurev-immunol-032713-120245 (2014). 24655296 10.1146/annurev-immunol-032713-120245

[4] Beischlag, T. V., Luis Morales, J., Hollingshead, B. D. & Perdew, G. H. The aryl hydrocarbon receptor complex and the control of gene expression. *Crit. Rev. Eukaryot. Gene Expr.* **18**(3), 207–250. 10.1615/critreveukargeneexpr.v18.i3.20 (2008). 18540824 10.1615/critreveukargeneexpr.v18.i3.20 PMC2583464

[5] Mulero-Navarro, S. & Fernandez-Salguero, P. M. New trends in aryl hydrocarbon receptor biology. *Front. Cell Dev. Biol.* **4**, 45. 10.3389/fcell.2016.00045 (2016). 27243009 10.3389/fcell.2016.00045 PMC4863130

[6] Gutierrez-Vazquez, C. & Quintana, F. J. Regulation of the immune response by the aryl hydrocarbon receptor. *Immunity* **48**(1), 19–33. 10.1016/j.immuni.2017.12.012 (2018). 29343438 10.1016/j.immuni.2017.12.012 PMC5777317

[7] Zhu, J. & JVdE, B. AHR and tryptophan metabolism: a collaborative dynamics of immune regulation. *Genes Immun.*10.1038/s41435-023-00235-6 (2024). 38104209 10.1038/s41435-023-00235-6

[8] Solvay, M. et al. Tryptophan depletion sensitizes the AHR pathway by increasing AHR expression and GCN2/LAT1-mediated kynurenine uptake, and potentiates induction of regulatory T lymphocytes. *J. Immunother. Cancer.*10.1136/jitc-2023-006728 (2023). 37344101 10.1136/jitc-2023-006728 PMC10314700

[9] Stockinger, B., Diaz, O. E. & Wincent, E. The influence of AHR on immune and tissue biology. *EMBO Mol. Med.* **16**(10), 2290–2298. 10.1038/s44321-024-00135-w (2024). 39242971 10.1038/s44321-024-00135-w PMC11473696

[10] Himmelfarb, J. Uremic toxicity, oxidative stress, and hemodialysis as renal replacement therapy. *Semin. Dial.* **22**(6), 636–643. 10.1111/j.1525-139X.2009.00659.x (2009). 20017834 10.1111/j.1525-139X.2009.00659.x

[11] Vaziri, N. D. Oxidative stress in uremia: Nature, mechanisms, and potential consequences. *Semin. Nephrol.* **24**(5), 469–473. 10.1016/j.semnephrol.2004.06.026 (2004). 15490413 10.1016/j.semnephrol.2004.06.026

[12] Meijers, B., Zadora, W. & Lowenstein, J. A historical perspective on uremia and uremic toxins. *Toxins (Basel)*10.3390/toxins16050227 (2024). 38787079 10.3390/toxins16050227 PMC11126090

[13] O’Brien, F. J. et al. Impaired tubular secretion of organic solutes in acute kidney injury. *Kidney360* **1**(8), 724–730. 10.34067/kid.0001632020 (2020). 35252876 10.34067/KID.0001632020 PMC8815732

[14] Tan, Y. Q. et al. Host/microbiota interactions-derived tryptophan metabolites modulate oxidative stress and inflammation via aryl hydrocarbon receptor signaling. *Free Radic. Biol. Med.* **184**, 30–41. 10.1016/j.freeradbiomed.2022.03.025 (2022). 35367341 10.1016/j.freeradbiomed.2022.03.025

[15] Hubbard, T. D., Murray, I. A. & Perdew, G. H. Indole and tryptophan metabolism: Endogenous and dietary routes to Ah receptor activation. *Drug Metab. Dispos.* **43**(10), 1522–1535. 10.1124/dmd.115.064246 (2015). 26041783 10.1124/dmd.115.064246 PMC4576673

[16] Zhang, L. et al. Development of a cell-seeded modified small intestinal submucosa for urethroplasty. *Heliyon* **2**(3), e00087. 10.1016/j.heliyon.2016.e00087 (2016). 27441265 10.1016/j.heliyon.2016.e00087 PMC4946073

[17] Lauriola, M. et al. Dietary protein intake and the tubular handling of indoxyl sulfate. *Nephrol. Dial. Transplant.*10.1093/ndt/gfae220 (2024). 10.1093/ndt/gfae220 39354683

[18] Csanaky, I. L., Lickteig, A. J. & Klaassen, C. D. Aryl hydrocarbon receptor (AhR) mediated short-term effects of 2,3,7,8-tetrachlorodibenzo-p-dioxin (TCDD) on bile acid homeostasis in mice. *Toxicol. Appl. Pharmacol.* **343**, 48–61. 10.1016/j.taap.2018.02.005 (2018). 29452137 10.1016/j.taap.2018.02.005 PMC5937549

[19] Nigam, S. K. What do drug transporters really do?. *Nat. Rev. Drug Discov.* **14**(1), 29–44. 10.1038/nrd4461 (2015). 25475361 10.1038/nrd4461 PMC4750486

[20] Nigam, S. K. & Bush, K. T. Uraemic syndrome of chronic kidney disease: Altered remote sensing and signalling. *Nat. Rev. Nephrol.* **15**(5), 301–316. 10.1038/s41581-019-0111-1 (2019). 30728454 10.1038/s41581-019-0111-1 PMC6619437

[21] Wang, H. et al. Genome-scale metabolic network reconstruction of model animals as a platform for translational research. *Proc. Natl. Acad. Sci. U. S. A.*10.1073/pnas.2102344118 (2021). 34282017 10.1073/pnas.2102344118 PMC8325244

[22] Sigurdsson, M. I., Jamshidi, N., Steingrimsson, E., Thiele, I. & Palsson, B. O. A detailed genome-wide reconstruction of mouse metabolism based on human Recon 1. *BMC Syst. Biol.* **4**, 140. 10.1186/1752-0509-4-140 (2010). 20959003 10.1186/1752-0509-4-140 PMC2978158

[23] Reed, J. L., Famili, I., Thiele, I. & Palsson, B. O. Towards multidimensional genome annotation. *Nat. Rev. Genet.* **7**(2), 130–141 (2006). 16418748 10.1038/nrg1769

[24] Mo, M. L., Jamshidi, N. & Palsson, B. O. A genome-scale, constraint-based approach to systems biology of human metabolism. *Mol. Biosyst.* **3**(9), 598–603. 10.1039/b705597h (2007). 17700859 10.1039/b705597h

[25] Brunk, E. et al. Recon3D enables a three-dimensional view of gene variation in human metabolism. *Nat. Biotechnol.* **36**(3), 272–281. 10.1038/nbt.4072 (2018). 29457794 10.1038/nbt.4072 PMC5840010

[26] Duarte, N. C. et al. Global reconstruction of the human metabolic network based on genomic and bibliomic data. *Proc. Natl. Acad. Sci. U. S. A.* **104**(6), 1777–1782. 10.1073/pnas.0610772104 (2007). 17267599 10.1073/pnas.0610772104 PMC1794290

[27] Thiele, I. et al. A community-driven global reconstruction of human metabolism. *Nat. Biotechnol.* **31**(5), 419–425. 10.1038/nbt.2488 (2013). 23455439 10.1038/nbt.2488 PMC3856361

[28] Bush, K. T., Singh, P. & Nigam, S. K. Gut-derived uremic toxin handling in vivo requires OAT-mediated tubular secretion in chronic kidney disease. *JCI. Insight*10.1172/jci.insight.133817 (2020). 32271169 10.1172/jci.insight.133817 PMC7205256

[29] Meijers, B. & Lowenstein, J. The evolving view of uremic toxicity. *Toxins (Basel)*10.3390/toxins14040274 (2022). 35448883 10.3390/toxins14040274 PMC9031373

[30] Meijers, B. K. & Evenepoel, P. The gut-kidney axis: Indoxyl sulfate, p-cresyl sulfate and CKD progression. *Nephrol. Dial. Transplant.* **26**(3), 759–761. 10.1093/ndt/gfq818 (2011). 21343587 10.1093/ndt/gfq818

[31] Jansen, J. et al. Remote sensing and signaling in kidney proximal tubules stimulates gut microbiome-derived organic anion secretion. *Proc. Natl. Acad. Sci. U. S. A.* **116**(32), 16105–16110. 10.1073/pnas.1821809116 (2019). 31341083 10.1073/pnas.1821809116 PMC6689987

[32] Ermakov, V. S., Granados, J. C. & Nigam, S. K. Remote effects of kidney drug transporter OAT1 on gut microbiome composition and urate homeostasis. *JCI. Insight*10.1172/jci.insight.172341 (2023). 37937647 10.1172/jci.insight.172341 PMC10721261

[33] Jamshidi, N. & Nigam, S. K. Drug transporters OAT1 and OAT3 have specific effects on multiple organs and gut microbiome as revealed by contextualized metabolic network reconstructions. *Sci. Rep.* **12**(1), 18308. 10.1038/s41598-022-21091-w (2022). 36316339 10.1038/s41598-022-21091-w PMC9622871

[34] Granados, J. C. et al. AHR is a master regulator of diverse pathways in endogenous metabolism. *Sci. Rep.*10.1038/s41598-022-20572-2 (2022). 36198709 10.1038/s41598-022-20572-2 PMC9534852

[35] Zelante, T. et al. Tryptophan catabolites from microbiota engage aryl hydrocarbon receptor and balance mucosal reactivity via interleukin-22. *Immunity* **39**(2), 372–385. 10.1016/j.immuni.2013.08.003 (2013). 23973224 10.1016/j.immuni.2013.08.003

[36] Puccetti, M. et al. Enteric formulated indole-3-carboxaldehyde targets the aryl hydrocarbon receptor for protection in a murine model of metabolic syndrome. *Int. J. Pharm.*10.1016/j.ijpharm.2021.120610 (2021). 33865951 10.1016/j.ijpharm.2021.120610

[37] Nigam, S. K. & Granados, J. C. OAT, OATP, and MRP drug transporters and the remote sensing and signaling theory. *Annu. Rev. Pharmacol. Toxicol.* **63**, 637–660. 10.1146/annurev-pharmtox-030322-084058 (2023). 36206988 10.1146/annurev-pharmtox-030322-084058

[38] Nigam, S. K. et al. Handling of drugs, metabolites, and uremic toxins by kidney proximal tubule drug transporters. *Clin. J. Am. Soc. Nephrol.* **10**(11), 2039–2049. 10.2215/CJN.02440314 (2015). 26490509 10.2215/CJN.02440314 PMC4633783

[39] Lowenstein, J. & Nigam, S. K. Uremic toxins in organ crosstalk. *Front. Med.* **8**, 592602. 10.3389/fmed.2021.592602 (2021). 10.3389/fmed.2021.592602 PMC8085272 33937275

[40] Vanholder, R., Nigam, S. K., Burtey, S. & Glorieux, G. What if not all metabolites from the uremic toxin generating pathways are toxic? A hypothesis. *Toxins (Basel).*10.3390/toxins14030221 (2022). 35324718 10.3390/toxins14030221 PMC8953523

[41] Yu, Z. et al. Pancreatic hormone insulin modulates organic anion transporter 1 in the kidney: regulation via remote sensing and signaling network. *AAPS J.*10.1208/s12248-022-00778-y (2023). 36627500 10.1208/s12248-022-00778-y PMC10695010

[42] Granados, J. C. et al. The kidney drug transporter OAT1 regulates gut microbiome-dependent host metabolism. *JCI Insight*10.1172/jci.insight.160437 (2023). 36692015 10.1172/jci.insight.160437 PMC9977316

[43] Lee, W. J. et al. Aryl hydrocarbon receptor deficiency attenuates oxidative stress-related mesangial cell activation and macrophage infiltration and extracellular matrix accumulation in diabetic nephropathy. *Antioxid. Redox. Signal.* **24**(4), 217–231. 10.1089/ars.2015.6310 (2016). 26415004 10.1089/ars.2015.6310

[44] Jamshidi, N., Nigam, K. B. & Nigam, S. K. Loss of the kidney urate transporter, urat1 leads to disrupted redox homeostasis in mice. *Antioxid. (Basel).*10.3390/antiox12030780 (2023). 10.3390/antiox12030780 PMC10045411 36979028

[45] Weiger, T. M. & Hermann, A. Cell proliferation, potassium channels, polyamines and their interactions: A mini review. *Amino. Acid.* **46**(3), 681–688. 10.1007/s00726-013-1536-7 (2014). 10.1007/s00726-013-1536-7 23820618

[46] Hu, J. et al. Exogenous spermine attenuates myocardial fibrosis in diabetic cardiomyopathy by inhibiting endoplasmic reticulum stress and the canonical Wnt signaling pathway. *Cell. Biol. Int.* **44**(8), 1660–1670. 10.1002/cbin.11360 (2020). 32304136 10.1002/cbin.11360

[47] Luo, D. et al. Metabolism of polyamines and kidney disease: a promising therapeutic target. *Kidney Dis. (Basel).* **9**(6), 469–484. 10.1159/000533296 (2023). 38089440 10.1159/000533296 PMC10712987

[48] Compagnone, A. et al. Polyamines modulate epithelial-to-mesenchymal transition. *Amino Acid.* **42**(2–3), 783–789. 10.1007/s00726-011-0995-y (2012). 10.1007/s00726-011-0995-y 21901470

[49] Russell, D. H. Clinical relevance of polyamines. *Crit. Rev. Clin. Lab. Sci.* **18**(3), 261–311. 10.3109/10408368209085073 (1983). 6339165 10.3109/10408368209085073

[50] Liu, J. R. et al. Gut microbiota-derived tryptophan metabolism mediates renal fibrosis by aryl hydrocarbon receptor signaling activation. *Cell Mol. Life Sci.* **78**(3), 909–922. 10.1007/s00018-020-03645-1 (2021). 32965514 10.1007/s00018-020-03645-1 PMC11073292

[51] Cao, G. et al. Intrarenal 1-methoxypyrene, an aryl hydrocarbon receptor agonist, mediates progressive tubulointerstitial fibrosis in mice. *Acta Pharmacol. Sin.* **43**(11), 2929–2945. 10.1038/s41401-022-00914-6 (2022). 35577910 10.1038/s41401-022-00914-6 PMC9622813

[52] Ng, H. Y. et al. Indoxyl sulfate downregulates expression of Mas receptor via OAT3/AhR/Stat3 pathway in proximal tubular cells. *PLoS ONE* **9**(3), e91517. 10.1371/journal.pone.0091517 (2014). 24614509 10.1371/journal.pone.0091517 PMC3948887

[53] Glorieux, G., Nigam, S. K., Vanholder, R. & Verbeke, F. Role of the microbiome in Gut-Heart-Kidney cross talk. *Circ. Res.* **132**(8), 1064–1083. 10.1161/CIRCRESAHA.123.321763 (2023). 37053274 10.1161/CIRCRESAHA.123.321763

[54] Walker, J. A. et al. Indoleamine 2,3-dioxygenase-1, a novel therapeutic target for post-vascular injury thrombosis in CKD. *J. Am. Soc. Nephrol.* **32**(11), 2834–2850. 10.1681/ASN.2020091310 (2021). 34716244 10.1681/ASN.2020091310 PMC8806102

[55] Mo, Y. et al. The aryl hydrocarbon receptor in chronic kidney disease: friend or foe?. *Front. Cell Dev. Biol.* **8**, 589752. 10.3389/fcell.2020.589752 (2020). 33415104 10.3389/fcell.2020.589752 PMC7784643

[56] Walker, J. A. et al. Temporal and tissue-specific activation of aryl hydrocarbon receptor in discrete mouse models of kidney disease. *Kidney Int.* **97**(3), 538–550. 10.1016/j.kint.2019.09.029 (2020). 31932072 10.1016/j.kint.2019.09.029 PMC9721455

[57] Heinrich, R., Rapoport, S. M. & Rapoport, T. A. Metabolic regulation and mathematical models. *Prog. Biophys. Mol. Biol.* **32**(1), 1–82 (1977). 343173

[58] Rapoport, T. A., Heinrich, R. & Rapoport, S. M. The regulatory principles of glycolysis in erythrocytes in vivo and in vitro. A minimal comprehensive model describing steady states, quasi-steady states and time-dependent processes. *Biochem. J.* **154**(2), 449–469 (1976). 132930 10.1042/bj1540449 PMC1172726

[59] Jamshidi, N. & Palsson, B. O. Systems biology of the human red blood cell. *Blood Cell. Mol. Dis.* **36**(2), 239–247. 10.1016/j.bcmd.2006.01.006 (2006). 10.1016/j.bcmd.2006.01.006 16533612

[60] Jamshidi, N. et al. Metabolome changes during in vivo red cell aging reveal disruption of key metabolic pathways. *iSci*10.1016/j.isci.2020.101630 (2020). 10.1016/j.isci.2020.101630 PMC7575880 33103072

[61] Chen, L. et al. NADPH production by the oxidative pentose-phosphate pathway supports folate metabolism. *Nat. Metab.* **1**, 404–415 (2019). 31058257 PMC6489125

[62] Lewis, C. A. et al. Tracing compartmentalized NADPH metabolism in the cytosol and mitochondria of mammalian cells. *Mol. Cell.* **55**(2), 253–263. 10.1016/j.molcel.2014.05.008 (2014). 24882210 10.1016/j.molcel.2014.05.008 PMC4106038

[63] Geenen, S. et al. Glutathione metabolism modeling: A mechanism for liver drug-robustness and a new biomarker strategy. *Biochim. Biophys. Acta* **1830**(10), 4943–4959. 10.1016/j.bbagen.2013.04.014 (2013). 23643929 10.1016/j.bbagen.2013.04.014

[64] Jamshidi, N., von Lohneysen, K., Soldau, K. & Friedman, J. S. The loss of peroxiredoxin 2 in mice disrupts the biochemical aging phenotype in erythrocytes. *iSci*10.1016/j.isci.2025.114608 (2026). 10.1016/j.isci.2025.114608 PMC12859242 41623488

[65] Ghosh, T., Philtron, D., Zhang, W., Kechris, K. & Ghosh, D. Reproducibility of mass spectrometry based metabolomics data. *BMC Bioinform.*10.1186/s12859-021-04336-9 (2021). 10.1186/s12859-021-04336-9 PMC8424977 34493210

[66] Boutros, P. C., Bielefeld, K. A., Pohjanvirta, R. & Harper, P. A. Dioxin-dependent and dioxin-independent gene batteries: Comparison of liver and kidney in AHR-null mice. *Toxicol. Sci.* **112**(1), 245–256. 10.1093/toxsci/kfp191 (2009). 19759094 10.1093/toxsci/kfp191 PMC2769058

[67] Jamshidi, N. & Raghunathan, A. Cell scale host-pathogen modeling: Another branch in the evolution of constraint-based methods. *Front. Microbiol.* **6**, 1032. 10.3389/fmicb.2015.01032 (2015). 26500611 10.3389/fmicb.2015.01032 PMC4594423

[68] Oh, Y. K., Palsson, B. O., Park, S. M., Schilling, C. H. & Mahadevan, R. Genome-scale reconstruction of metabolic network in *Bacillus subtilis* based on high-throughput phenotyping and gene essentiality data. *J. Biol. Chem.* **282**(39), 28791–28799. 10.1074/jbc.M703759200 (2007). 17573341 10.1074/jbc.M703759200

[69] Becker, S. A. & Palsson, B. O. Context-specific metabolic networks are consistent with experiments. *PLoS Comput. Biol.* **4**(5), e1000082. 10.1371/journal.pcbi.1000082 (2008). 18483554 10.1371/journal.pcbi.1000082 PMC2366062

[70] Krzywinski, M. et al. Circos: An information aesthetic for comparative genomics. *Genome Res.* **19**(9), 1639–1645. 10.1101/gr.092759.109 (2009). 19541911 10.1101/gr.092759.109 PMC2752132

[71] Ebrahim, A., Lerman, J. A., Palsson, B. O. & Hyduke, D. R. COBRApy: COnstraints-based reconstruction and analysis for python. *BMC Syst. Biol.* **7**, 74. 10.1186/1752-0509-7-74 (2013). 23927696 10.1186/1752-0509-7-74 PMC3751080

[72] Schellenberger, J. et al. Quantitative prediction of cellular metabolism with constraint-based models: The COBRA Toolbox v2.0. *Nat. Protoc.* **6**(9), 1290–1307 (2011). 21886097 10.1038/nprot.2011.308 PMC3319681

[73] King, Z. A. et al. Escher: A web application for building, sharing, and embedding data-rich visualizations of biological pathways. *PLoS Comput. Biol.* **11**(8), e1004321. 10.1371/journal.pcbi.1004321 (2015). 26313928 10.1371/journal.pcbi.1004321 PMC4552468

