# Supplementary figures and images for: Aryl hydrocarbon receptor in the kidney regulates metabolic cross-talk with the liver and gut microbiome
Source: Sci Rep. 2026 Mar 24;16:14879. doi: 10.1038/s41598-026-44083-6 (PMC13168485; doi:10.1038/s41598-026-44083-6)

Supplemental Figure 1

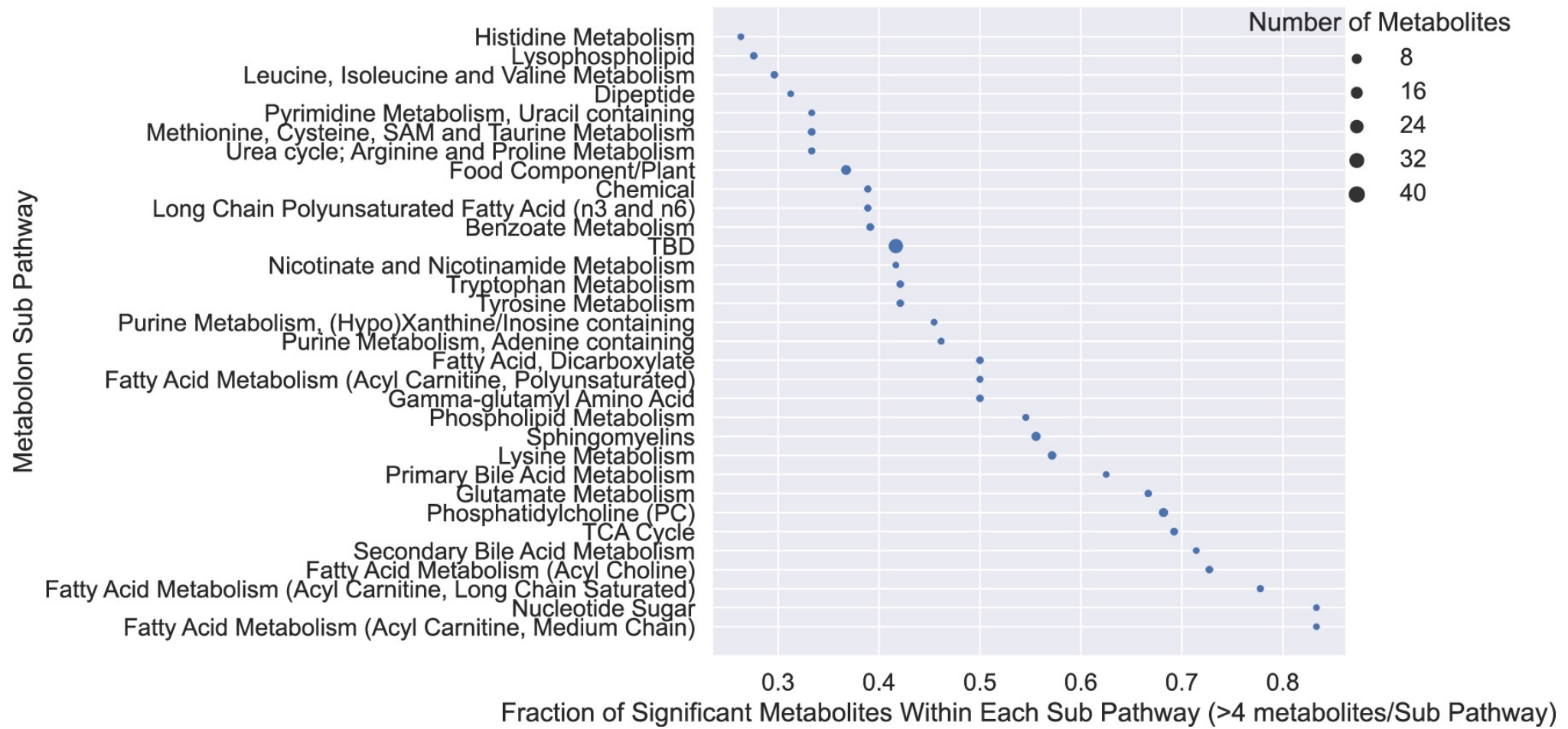

Supplemental Figure 2

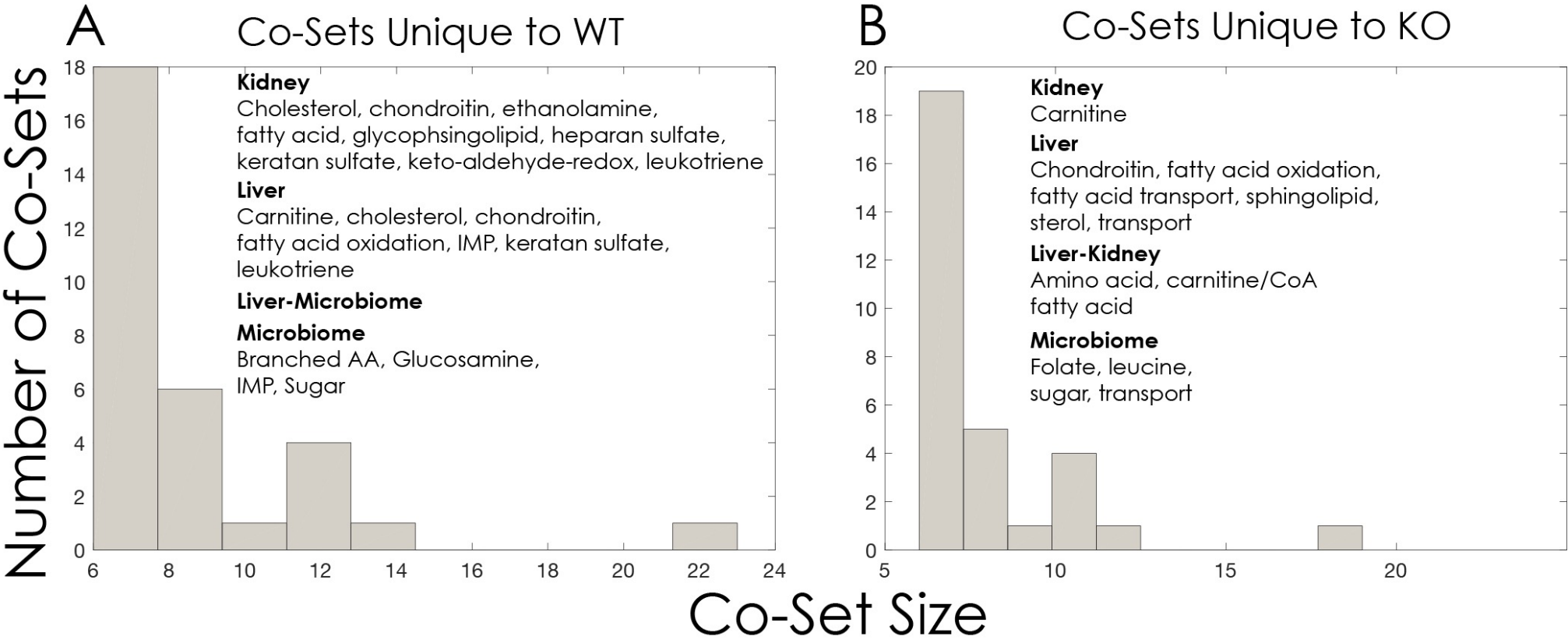

## Supplemental Figure 3

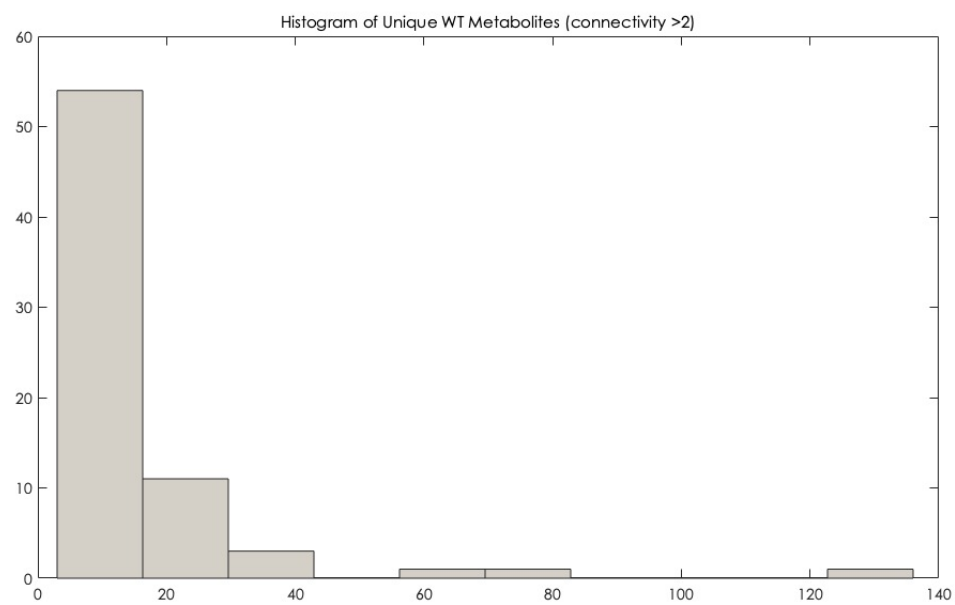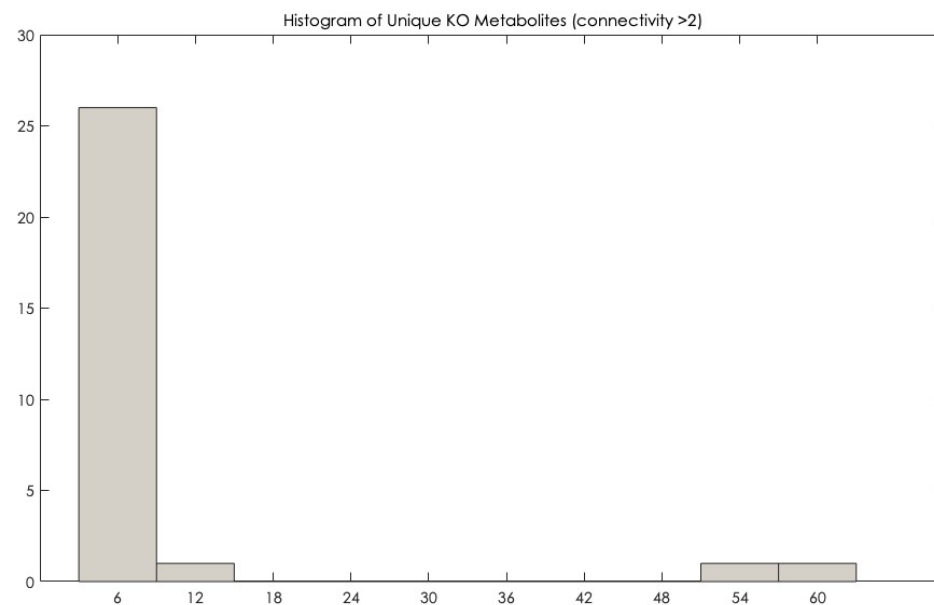

Supplement: Supplementary file 1 — Supplementary Information 1. [file 41598_2026_44083_MOESM1_ESM.pdf]
