# Supplementary material for: Aryl hydrocarbon receptor in the kidney regulates metabolic cross-talk with the liver and gut microbiome
Source: Sci Rep. 2026 Mar 24;16:14879. doi: 10.1038/s41598-026-44083-6 (PMC13168485; doi:10.1038/s41598-026-44083-6)
Supplement: Supplementary file 2 — Supplementary Information 2. [file 41598_2026_44083_MOESM2_ESM.docx]

**Supplemental Legends**

**Supplemental Figure 1.**

Broad, systemic changes in the intracellular kidney metabolome. We evaluated the intracellular kidney metabolome and found that there are alterations across a broad spectrum of metabolic processes in AHR KO. However, these are largely distinct from the changes seen in plasma. Shown are intracellular kidney metabolomics in AHR knockout versus wildtype mice. Sub-system enrichment is summarized for significantly altered metabolites in AHR knockout mice comparted to wildtype. When metabolites are classified according to sub-systems, there is broad involvement of central as well as more peripheral metabolic processes.

**Supplemental Figure 2.**

Statistical summary of functional pathways in WT and AHR KO multi-tissue, multi-organism models.

A) and B) histograms summarizing the co-sets (correlated reaction sets) unique to the WT and KO MOMRs, respectively (also corresponding to Tables 2 and 3, respectively). Interestingly the relative size and distribution of the co-sets are similar in WT and KO conditions, reflecting consistency in general metabolic characteristics (pathway length, metabolic processes, etc.), but with differences related to correlations among reactions involving different substrates and products.

**Supplemental Figure 3.**

Comparison of WT and KO MOMR models and then selecting metabolites with highest connectivity identified 8 metabolites in the kidney (in the cytosol or mitochondria intracellular compartments). These metabolites are all closely connected with the urea cycle.

**Supplemental Table 1.**

Wildtype and knockout mouse phenotypic data.

**Supplemental Table 2.**

Metabolite volcano plot with selected outliers

**Supplemental Table 3.**

Discordant metabolites

**Supplemental Table 4.**

Wildtype cosets

**Supplemental Table 5.**

AHR knockout cosets
